# AKT/AMPK-mediated phosphorylation of TBC1D4 disrupts the interaction with insulin-regulated aminopeptidase

**DOI:** 10.1016/j.jbc.2021.100637

**Published:** 2021-04-16

**Authors:** Samaneh Eickelschulte, Sonja Hartwig, Ben Leiser, Stefan Lehr, Viola Joschko, Manopriya Chokkalingam, Alexandra Chadt, Hadi Al-Hasani

**Affiliations:** 1 Medical Faculty, Institute of Clinical Biochemistry and Pathobiochemistry, German Diabetes Center, Leibniz Center for Diabetes Research at Heinrich Heine University, Düsseldorf, Germany; 2 German Center for Diabetes Research (DZD), Partner Düsseldorf, München-Neuherberg, Germany

**Keywords:** GTPase-activating protein (GAP), protein phosphorylation, glucose transporter type 4 (GLUT4), Rab10, Akt PKB, insulin-regulated aminopeptidase (IRAP), AICAR, 5-aminoimidazole-4-carboxamide 1 β-D-ribonucleoside, IMAC, immobilized metal affinity chromatography, IRAP, insulin-regulated aminopeptidase, PTB, phosphotyrosine-binding domain, Rab, Ras-related proteins in brain, RabGAP, Rab GTPase-activating protein

## Abstract

TBC1D4 is a 160 kDa multidomain Rab GTPase-activating protein (RabGAP) and a downstream target of the insulin- and contraction-activated kinases AKT and AMPK. Phosphorylation of TBC1D4 has been linked to translocation of GLUT4 from storage vesicles (GSVs) to the cell surface. However, its impact on enzymatic activity is not well understood, as previous studies mostly investigated the truncated GAP domain lacking the known phosphorylation sites. In the present study, we expressed and purified recombinant full-length TBC1D4 using a baculovirus system. Size-exclusion chromatography and coimmunoprecipitation experiments revealed that full-length TBC1D4 forms oligomers of ∼600 kDa. Compared with the truncated GAP domain, full-length TBC1D4 displayed similar substrate specificity, but had a markedly higher specific GAP activity toward Rab10. Using high-resolution mass spectrometry, we mapped 19 Ser/Thr phosphorylation sites in TBC1D4. We determined Michaelis–Menten kinetics using *in vitro* phosphorylation assays with purified kinases and stable isotope-labeled γ-[^18^O_4_]-ATP. These data revealed that Ser^324^ (K_M_ ∼6 μM) and Thr^649^ (K_M_ ∼25 μM) were preferential sites for phosphorylation by AKT, whereas Ser^348^, Ser^577^, Ser^595^ (K_M_ ∼10 μM), Ser^711^ (K_M_ ∼79 μM), and Ser^764^ were found to be preferred targets for AMPK. Phosphorylation of TBC1D4 by AKT or AMPK did not alter the intrinsic RabGAP activity, but did disrupt interaction with insulin-regulated aminopeptidase (IRAP), a resident protein of GSVs implicated in GLUT4 trafficking. These findings provide evidence that insulin and contraction may regulate TBC1D4 function primarily by disrupting the recruitment of the RabGAP to GLUT4 vesicles.

In skeletal muscle, stimulation by both insulin and contraction triggers an increase in glucose uptake from the blood stream through a rapid and reversible redistribution of GLUT4 from GLUT4 transporter storage vesicles (GSVs) to the cell surface (1, 2). Phosphorylation cascades transmitted *via* AKT or AMPK kinases as well as Rab (Ras-related proteins in brain) GTPases in their active GTP-bound form are thought to be responsible for relieving the intracellular retention of GLUT4 in the basal state (3, 4). The Rab GTPase-activating proteins (RabGAPs) TBC1D1 and TBC1D4 (also known as AS160) are downstream targets of AKT and AMPK kinases, and phosphorylation of these two proteins is associated with enhanced translocation of GLUT4 to the plasma membrane in response to insulin and contraction (5, 6). TBC1D1 and TBC1D4 share a similar domain structure and are comprised of two N-terminal phosphotyrosine-binding (PTB) domains, a calmodulin-binding domain (CB), and a GTPase-activating (Tre-2/Bub2/Cdc16-or GAP) domain toward the C-terminal end (3, 7). Previous studies show that both TBC1D1 and TBC1D4 are phosphorylated at multiple serine/threonine residues by AKT and AMPK *in vitro* (7, 8) and in response to insulin stimulation and exercise *in vivo* (5, 9). The majority of the known phosphorylation sites are localized within the second PTB domain and the region between the PTB domains and the C-terminal GAP domain. The mechanism of TBC1D1 and TBC1D4 regulation through AKT and AMPK phosphorylation in GLUT4 trafficking has been of particular interest for many years. Previous studies suggested a point of convergence for AKT and AMPK signaling through elevated insulin-stimulated phosphorylation of TBC1D4 after contraction (10, 11) and in particular AMPK activation (12, 13, 14). Due to the fact that all previous studies utilized truncated GAP domain of TBC1D4, the molecular function of the phosphorylation sites remains largely unknown. In this study, we focused on the regulation of recombinant full-length TBC1D4 through AKT-and AMPK-dependent phosphorylation and investigate the temporal and spatial pattern of TBC1D4 phosphorylation in details. After mapping TBC1D4 phosphorylation sites, our results show that AKT and AMPK have different affinity toward their target phosphorylation sites. In addition, phosphorylation of specific sites in TBC1D4 may influence phosphorylation of other sites, which may explain in part the insulin-sensitizing effect of muscle contraction.

## Results

### Expression, purification, and oligomerization of recombinant His_6_-TBC1D4

**Figure 1:**
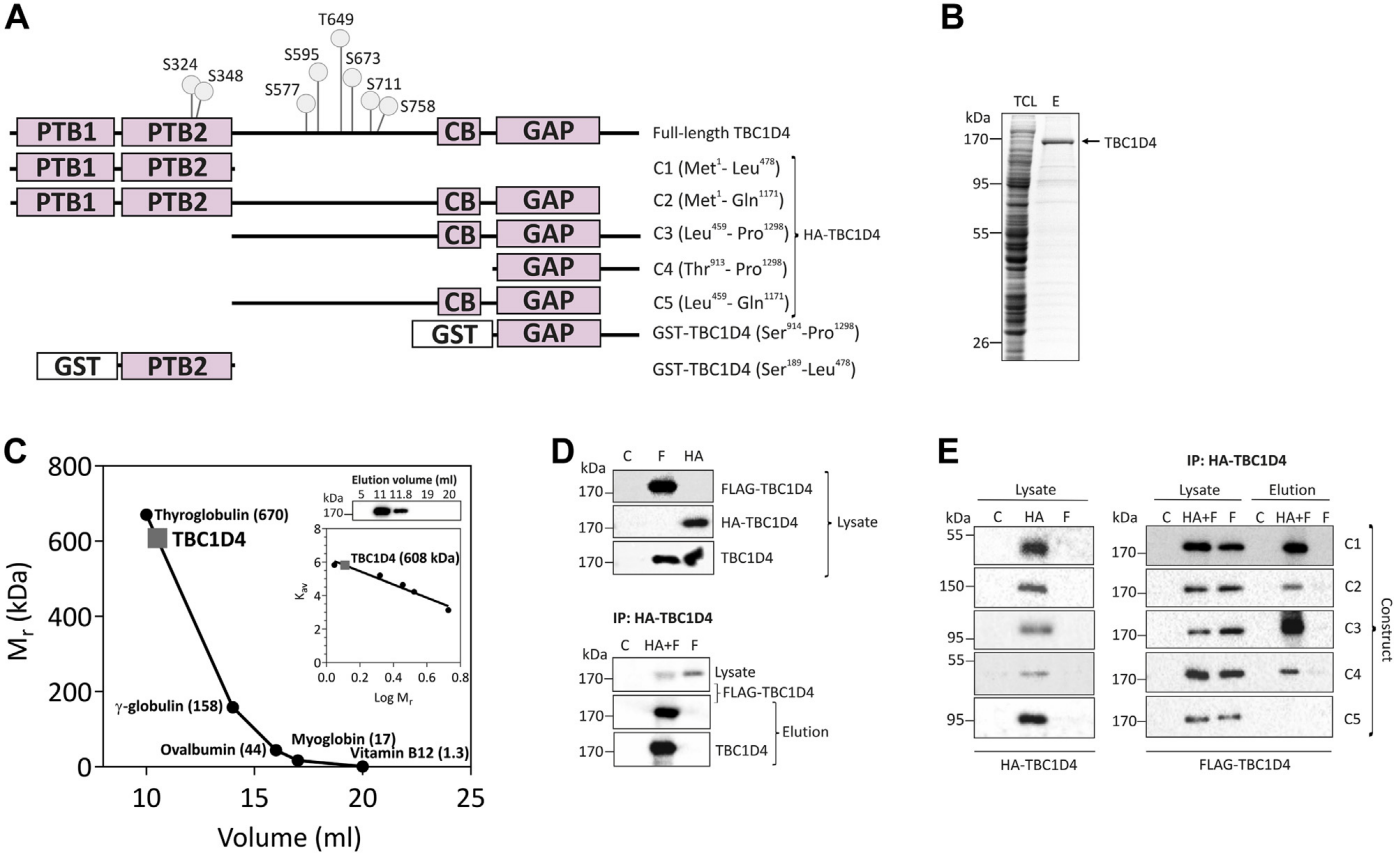
**Domain structure, expression, and oligomerization of recombinant full-length mouse TBC1D4 protein.***A*, schematic representation of the full-length 1298-amino-acid isoform of the murine TBC1D4, HA-TBC1D4 constructs (C1–C5), and truncated GST-tagged constructs (GST-PTB2 and GST-GAP) used in this study. The reported phosphorylation sites (at least in two independent studies (3, 8, 11, 14, 18, 19, 30, 31, 32, 34, 35)) for AKT and AMPK, Ser^324^, Ser^348^, Ser^577^, Ser^595^, Thr^649^, Ser^673^, Ser^711^, and Ser^758^ (corresponding to human sites Ser^318^, Ser^341^, Ser^570^, Ser^588^, Thr^642^, Ser^666^, Ser^704^, and Ser^751^) are indicated. *B*, Coomassie-stained SDS-PAGE of total *Sf*9 cell lysate (TCL) and recombinant His_6_-purified TBC1D4 protein after elusion (E) using affinity Ni-NTA chromatography. *C*, size-exclusion chromatography profile of purified TBC1D4. *Inset*: Western blot of selected SEC fractions using TBC1D4 antibodies and estimation of apparent molecular mass through linear regression of log (M_r_) and partition coefficient (K_*av*_). *D*, co-immunoprecipitation of HA-TBC1D4 and 3xFLAG-TBC1D4. Cleared lysates from HEK293 cells coexpressing full-length HA-or 3xFLAG tagged-TBC1D4 or both, respectively, were incubated with magnetic beads coated with anti-HA monoclonal antibodies as described under “Experimental procedures.” Samples were separated by SDS-PAGE and coprecipitated FLAG-TBC1D4 was analyzed *via* western blot analysis using nontransfected HEK293 cells (C), HEK293 cells expressing 3xFLAG-TBC1D4 exclusively (F) and HA-TBC1D4 (HA) as controls. *E*, *left panel*: cleared lysates from HEK293 cells (C), HEK293 cells expressing HA-TBC1D4 constructs (HA) and full-length 3xFLAG tagged-TBC1D4 (F), *right panel*: co-immunoprecipitation of HA-TBC1D4 constructs and full-length 3xFLAG-TBC1D4.

We used the baculovirus expression system to express full-length recombinant TBC1D4 protein. The cDNA for the long isoform (1298 aa) of the murine *Tbc1d4* gene was cloned into the transfer vector pAcSG2-6xHis to create a baculovirus as described previously (7). The construct for His_6_-TBC1D4 includes two N-terminal PTB domains, a central CB domain, and a C-terminal GAP domain (Fig. 1*A*). Baculovirus-infected *Sf*9 cells were cultured, harvested, and the recombinant protein was purified as described under “Experimental procedures”. The yield of TBC1D4 after IMAC was approximately 1 mg/10^9^ *Sf*9 cells with a purity of ∼80% as determined by densitometry of Coomassie-stained gels. In SDS-PAGE the purified protein appeared as a single band with an apparent M_r_ of ∼170 kDa (Fig. 1*B*). In contrast, using size-exclusion chromatography (SEC), the apparent molecular mass of the purified protein corresponded to 608 kDa (Fig. 1*C* and Fig. S1). To explore possible oligomerization of TBC1D4, we conducted co-immunoprecipitation experiments using lysates from HEK293 cells expressing either full-length HA-tagged TBC1D4, FLAG-tagged TBC1D4 (Fig. 1*D*, upper panel), or both tagged proteins. As shown in Figure 1*D* (lower panel), immunoprecipitation of HA-TBC1D4 with HA-antibodies resulted in coprecipitation of both HA-TBC1D4 and FLAG-TBC1D4. To map the interacting domains of TBC1D4, we coexpressed FLAG-tagged full-length TBC1D4 and different HA-tagged domain constructs of TBC1D4 (Fig. 1*A*) and analyzed protein–protein interactions with immunoprecipitation using HA- and FLAG antibodies. As illustrated in Figure 1*E*, FLAG-tagged TBC1D4 coprecipitated with the HA-tagged N-terminal PTB domains (C1 and C2; Fig. 1*A*), the residual CB/GAP domains with the C-terminal tail of TBC1D4 (C3 and C4; Fig. 1*A*) but not with the protein lacking both PTB domains and the 127 amino-acid-spanning C-terminal tail (C5; Fig. 1*A*). We further investigated structural features of TBC1D4 complexes by limited proteolysis using low concentration of trypsin and analysis of partial digestion products by mass spectrometry. Interestingly, rapid fragmentation occurred predominantly at tryptic sites close to two phosphorylation sites in TBC1D4, Ser^348^ and Ser^577^ (Fig. 1*A*; Fig. S2).

### GAP-activity of full-length TBC1D4 protein toward Rab10

**Figure 2:**
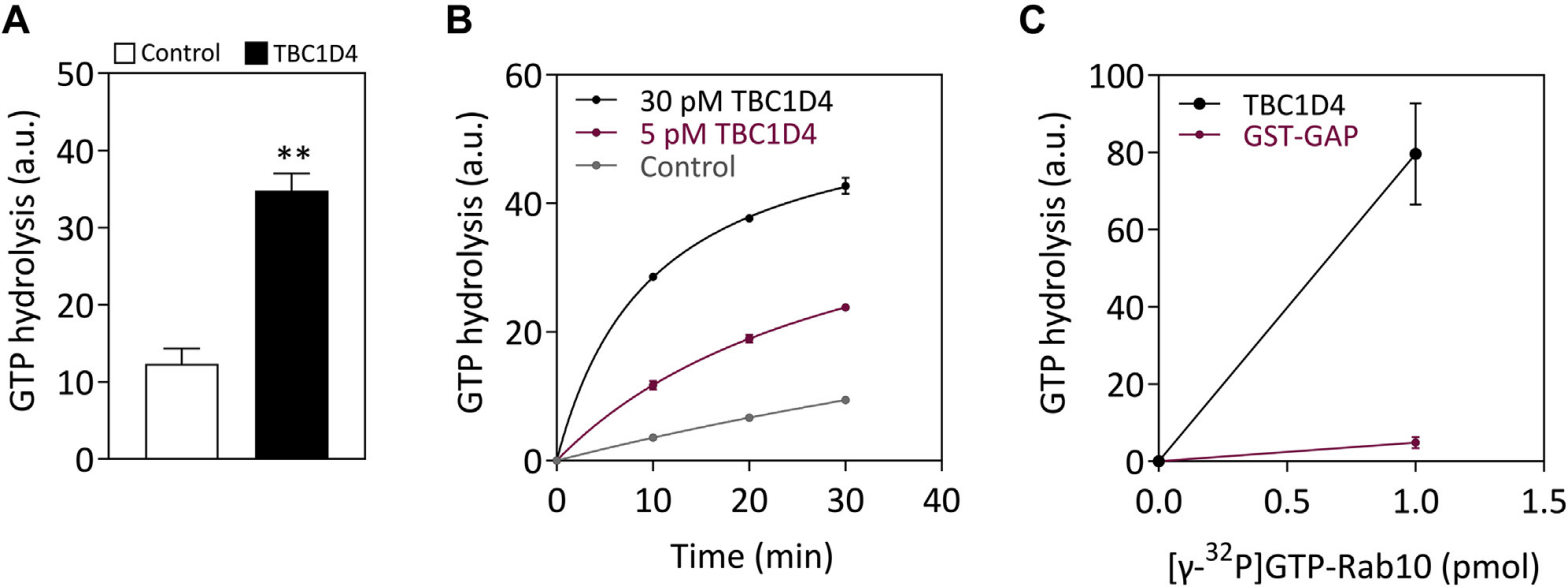
**Analysis of TBC1D4 RabGAP activity *in vitro*.***A*, RabGAP assays were performed toward Rab10 in the presence of full-length TBC1D4 protein. Affinity-purified GST-Rab10 (0.6–1 pmol) loaded with [γ-^32^P]GTP was incubated in the absence or presence of 3 pmol of purified TBC1D4 as described under “Experimental procedures.” After 30 min at room temperature (RT), aliquots were filtrated through activated charcoal and radioactive [^32^P]phosphate was determined by scintillation counting. Data represent mean values ± SEM from three independent experiments. ∗∗*p* = 0.0017, two-tailed unpaired *t*-test. *B*, purified TBC1D4 was incubated at different concentrations with [γ-^32^P]GTP loaded GST-Rab10 (2–6 pmol) at RT. At different time points aliquots were removed, filtrated through activated charcoal, and radioactive [^32^P]phosphate was determined by scintillation counting. *C*, 60 pmol of purified truncated GAP domain (GST-GAP) and 2 pmol of full-length TBC1D4 protein were incubated with [γ-^32^P]GTP-loaded (0.6–1 pmol) GST-Rab10 for 30 min at RT, and the amount of released [^32^P]phosphate was determined by scintillation counting. Phosphate production resulting from the endogenous GTP hydrolysis activity of Rab10 was subtracted. Data adjusted to the concentration of full-length TBC1D4 and truncated GAP domain and represent mean values ± SEM from three independent experiments.

We investigated the catalytic activity of TBC1D4 and performed RabGAP assays as described in “Experimental procedures.” The GTPase Rab10 has been previously identified as major substrate for the truncated GAP domain of TBC1D4 (15, 16). We therefore determined the GTPase activity of full-length TBC1D4 protein toward recombinant Rab10 *in vitro*. Purified GST-Rab10 was loaded with [γ-^32^P]GTP, incubated with purified full-length TBC1D4 protein, and the rate of [^32^P]phosphate release resulting from hydrolysis of [γ-^32^P]GTP was measured as described previously (7). Figure 2, *A* and *B* illustrate that purified GST-Rab10 exhibited some endogenous GTPase activity, but the initial velocity of GTP hydrolysis was substantially increased in the presence of full-length TBC1D4. Similar results were obtained for Rab8a (data not shown), consistent with previous studies that identified main Rab substrates using recombinant GAP domain expressed and purified from *E. coli* (4, 17). We next compared the specific GAP activities of the full-length TBC1D4 purified from *Sf9* cells and the truncated GAP domain expressed and purified as GST-tagged fusion protein in *E. coli* toward Rab10. Both GAPs were incubated with γ[^32^P]GTP-loaded GST-Rab10 and the amount of released [^32^P]phosphate was measured. Compared with the truncated GAP domain, the full-length protein displayed a two orders of magnitude higher activity toward Rab10 as substrate (Fig. 2*C*).

### Mapping AKT- and AMPK-dependent phosphorylation sites in TBC1D4

Previous studies have demonstrated that TBC1D4 is phosphorylated *in vivo* at 16 Ser/Thr phosphorylation sites in response to insulin stimulation, muscle contraction, and simulation with the AMPK activator AICAR (1, 3, 8, 14, 18, 19, 20) (Fig. S3). In order to experimentally validate the presumed role of AKT and AMPK as upstream kinases, we conducted *in vitro* phosphorylation reactions using purified kinases and employed quantitative high-resolution mass spectrometry to map the TBC1D4 phosphorylation sites. Full-length TBC1D4 purified from baculovirus-infected *Sf9* cells exhibited phosphorylation of 19 different Ser/Thr residues, including several sites previously identified in mammalian cells (Table 1; Table S1). We therefore used dephosphorylated recombinant TBC1D4 and performed *in vitro* kinase assays using purified AKT or AMPK and stable isotope-labeled [γ-^18^O_4_]ATP as described in “Experimental procedures.” Mass spectrometric analysis of tryptic TBC1D4 peptides revealed Ser^324^ and Thr^649^ as predominant phosphorylation targets for AKT, Ser^348^, Ser^577^, Ser^711^, and Ser^764^ as main target sites for AMPK, and Ser^595^ as a target site for both AKT and AMPK (Table 1; Table S1).

**Table 1:** TBC1D4 phosphorylation sites detected by mass spectrometry

Residue position^a^	Phosphopeptide^b^	Prephosphorylated site ([^16^O]-label)	AKT/AMPK-phosphorylated site ([^18^O]-label)	References
Ser^258^	GGDPGDEMGVLEVEp**S**PVSPDDSLPE	X	–	–
Ser^261^	GGDPGDEMGVLEVESPVp**S**PDDSLPE	X		
Ser^276^	ADGTVNp**S**P	X	–	Contraction (20)
Ser^324^	EFRSRCSp**S**VTGVMQK	–	AKT	AKT/exercise (3, 8, 18, 19, 20, 30)
Ser^348^	HAp**S**APSHVQPSDSE	X	AMPK	Insulin/exercise (8, 18)
Ser^377^	FEINLIp**S**PDT	X	–	–
Ser^492^	HLp**S**SLTDNEQADIFE	X	–	–
Ser^577^	SLTSp**S**LENIFS	X	AMPK	AKT/insulin (3, 8)
Ser^595^	GRLGp**S**MDSFE	X	AKT, AMPK	Insulin/exercise (3, 8, 18, 19, 20)
Ser^598^	GRLGSMDp**S**FE	X	–	Contraction (20)
Ser^604^	ANp**S**LASEKDFSPGDp**S**PPGTPPASPLSSAWHAFPEEDSDSPQF	–	–	Exercise (33)
Ser^616^				Contraction (20)
Thr^649^	RAHp**T**FSHPPSSSR	X	AKT	AKT/insulin (3, 14, 18, 20, 31)
Ser^673^	AHGLRp**S**PLL	X		Insulin/IGF–1 (8, 35)
Ser^680^	QSp**S**SEQCSIVPSA	X		AICAR (20)
Ser^711^	ESNSSCSLPSLHTSFp**S**APSFTAPSFL	X	AMPK	Insulin/exercise (14, 18, 20, 30, 32)
Ser^758^	EGRKRTSp**S**TCSNESL	–	–	Insulin/exercise (3, 8, 18, 19, 34)
Ser^761^	TSSTCp**S**NESLNAGGTPVTP	X	–	AICAR (20)
Ser^764^	TSSTCSNEp**S**LNAGGTPVTP	–	AMPK	AICAR (20)
Ser^789^	VAp**S**PVNKSPSAMQQQ	X	–	Contraction (20)
Ser^794^	VASPVNKp**S**PSAMQQQ	X	–	–
Ser^817^	DGLDRTELLPLSPLp**S**PTMEEEPLIIFLSGDEDTE	X	–	–
Ser^1135^	VALSLLSp**S**QEALIME	–	–	AICAR (20)

Purified full-length TBC1D4 expressed in *Sf9* cells was separated by SDS-PAGE, and tryptic phosphopeptides from excised Coomassie-stained protein bands were analyzed using LC/MS mass spectrometry as described in “Experimental procedures.” In parallel, enzymatically dephosphorylated purified TBC1D4 was rephosphorylated by AKT and AMPK *in vitro* in the presence of isotopically labeled γ[^18^O_4_]ATP for 5 to 10 min, separated by SDS-PAGE, excised, digested with trypsin, and the resulting [^18^O]-labeled phosphopeptides were also analyzed by LC/MS mass spectrometry. Sequences of phosphopeptides and phosphorylated Ser/Thr residues are indicated. Prephosphorylated phosphosites were identified to contain [^16^O_3_]phosphoryl groups, direct *in vitro* targets of AKT and/or AMPK were identified by containing [^18^O_3_]phosphoryl groups. References refer to previously reported upstream kinase and/or stimulus.

a Accession Q8BYJ6.

b pS, pT denote phosphorylated Ser/Thr residue; underlined residues indicate the presence within consensus motifs for AKT and AMPK, *RXRXXS/T*, *RXXS/T*. Listed are prototypic tryptic peptides, additional information on detected sequences is listed in Table S1. The mass spectrometry proteomics data have been deposited to the ProteomeXchange Consortium *via* the PRIDE (49) partner repository with the dataset identifiers PXD024621 and PXD024645.

### AKT- and AMPK-dependent phosphorylation kinetics

**Figure 3:**
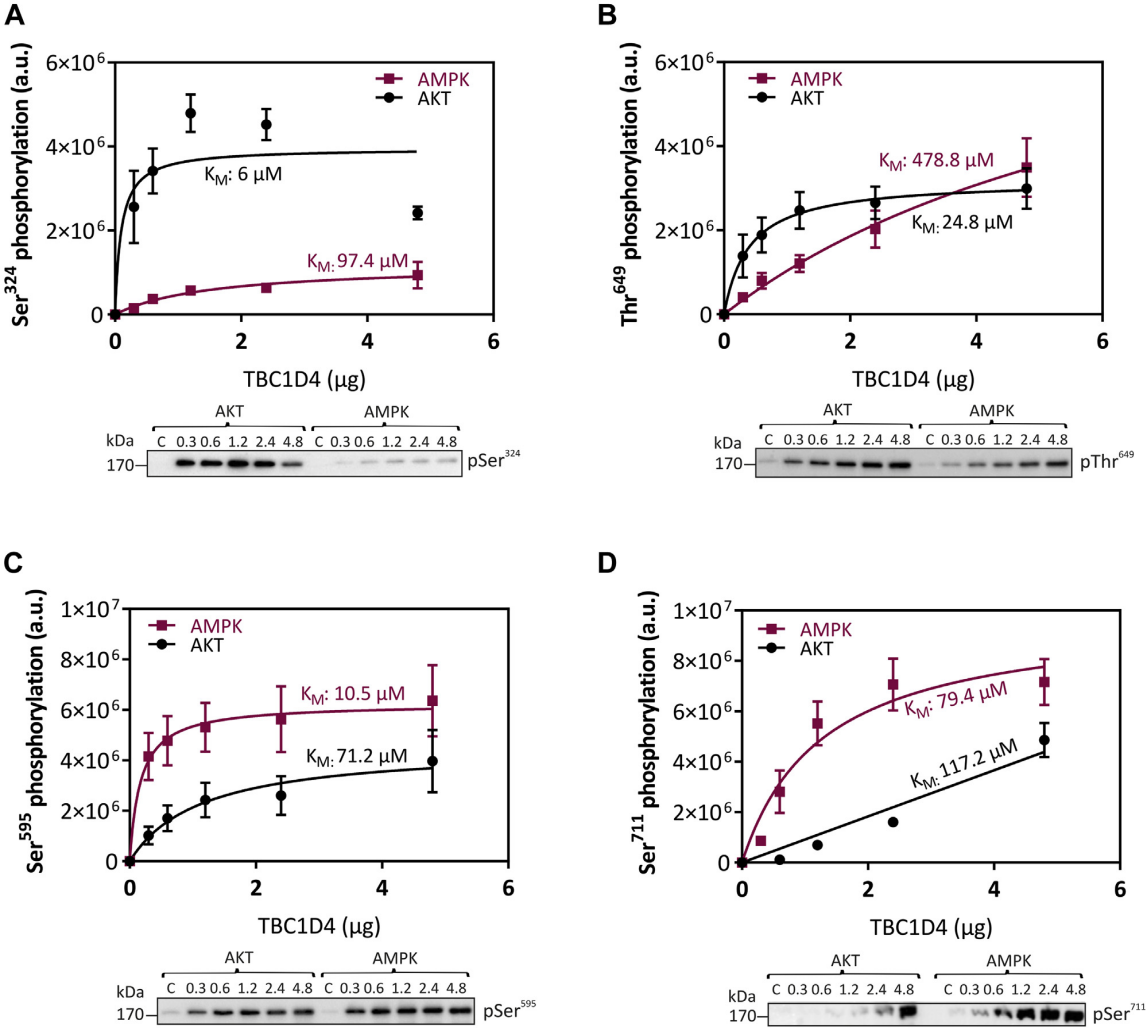
**Kinetics of TBC1D4 phosphorylation by AKT and AMPK *in vitro*.** Purified AKT and AMPK kinases were used to phosphorylate purified full-length TBC1D4 protein (between 0.3 and 4.8 μg) for 5 min at RT. Phosphorylation of TBC1D4 was confirmed using phosphosite-specific antibodies against *A*, Ser^324^, *B*, Thr^649^, *C*, Ser^595^, and *D*, Ser^711^, respectively, and the data were then subjected to nonlinear curve fitting to Michaelis–Menten kinetics using GraphPad Prism Software. Data represent mean values ± SEM from four independent experiments.

We studied phosphorylation of TBC1D4 in more details and conducted kinetic analyses of the phosphorylation reactions. Increasing concentrations of purified TBC1D4 protein were incubated with either purified AKT or AMPK kinase and phosphorylation of TBC1D4 was quantified by western blot analysis using phosphosite-specific antibodies. Initial velocities for each phosphorylation site including pSer^324^, pSer^595^, pThr^649^, and pSer^711^ were then subjected to nonlinear curve fitting to Michaelis–Menten kinetics to determine apparent K_M_ and V_max_ values for the individual phosphorylation sites. As shown in Figure 3, all sites analyzed were phosphorylated by either AKT or AMPK, albeit at substantially different degrees. AKT showed strong preference for pSer^324^ and pThr^649^ (K_M_ pSer^324^: 6 μM; K_M_ pThr^649^: 24.8 μM; Fig. 3, *A* and *B*) where corresponding K_M_ values for AMPK were about 20 times higher (K_M_ pSer^324^: 97.4 μM; K_M_ pThr^649^: 478.8 μM; Fig. 3, *A* and *B*). Conversely, the AMPK K_M_ value for phosphorylation of Ser^595^ was sevenfold lower than that for AKT (10.5 μM *versus* 71.2 μM; Fig. 3*C*), whereas K_M_ values obtained for phosphorylation of Ser^711^ by AKT (79.4 μM) and AMPK (117.2 μM) were similar (Fig. 3*D*). Relative V_max_ values for the phosphorylation sites were mostly in the same order of magnitude for AKT and AMPK (Fig. S4).

### Impact of phosphorylation on the TBC1D4 RabGAP activity

**Figure 4:**
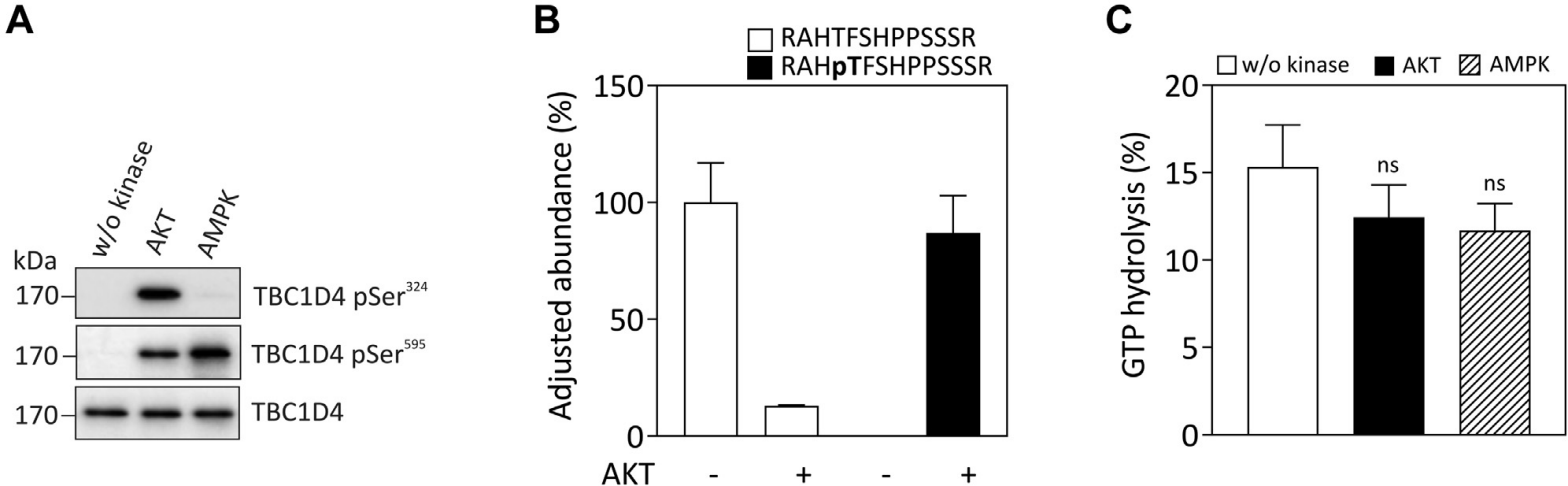
**Impact of the TBC1D4 phosphorylation state on RabGAP activity *in vitro*.***A*, 3 pmol of purified full-lengthTBC1D4 protein was phosphorylated for 30 min at RT with purified AKT or AMPK, respectively. TBC1D4 phosphorylation was confirmed by western blot analysis using phosphosite-specific antibodies against pSer^324^ and pSer^595^. *B*, adjusted abundance of a peptide with and without Thr^649^ modification from TBC1D4 protein in a buffer containing 2 mM labeled [γ-^18^O_4_]ATP, AKT+[γ-^18^O_4_]ATP treatments has been shown. The amount of phosphorylation by [γ-^18^O_4_]ATP + AKT was determined by considering the nonphosphorylated peptide’s fractional amounts in [γ-^18^O_4_]ATP and [γ-^18^O_4_]ATP + AKT treatments. The standard errors from the mean of peptide’s fractional amounts were linear transformed for the respective treatments. *C*, nonphosphorylated TBC1D4, AKT-phosphorylated, or AMPK-phosphorylated TBC1D4, respectively, were incubated with purified [γ-^32^P]GTP-loaded GST-Rab10 (0.6–1 pmol) for 30 min at RT, and the amount of released [^32^P]phosphate was determined by scintillation counting as described under “Experimental procedures.” Data represent mean values ± SEM from five independent experiments; ns, not significant, one-way ANOVA.

Mutation of putative Ser/Thr phosphorylation sites to alanine, in particular at Ser^324^, Ser^595^, Thr^649^, and Ser^758^ (Ser^318^, Ser^588^, Thr^642^, and Ser^751^ in the human sequence) in TBC1D4, and overexpression of the mutant protein (“4P”) have been shown to impair GLUT4 translocation in 3T3-L1 adipocytes, suggesting an inhibitory effect of the TBC1D4 phosphorylation state on its RabGAP activity (3). To directly investigate the impact of phosphorylation on the RabGAP activity *in vitro*, we conducted RabGAP assays with purified full-length TBC1D4 protein, previously phosphorylated by AKT or AMPK using [γ-^32^P]GTP-loaded Rab10 as substrate. TBC1D4 was phosphorylated *in vitro* for 30 min by AKT or AMPK to achieve saturation of the phosphorylation reaction. Subsequent western blot analysis using phosphosite-specific antibodies revealed markedly increased immunoreactivity for pSer^324^ for AKT and in addition pSer^595^ for AMPK (Fig. 4*A*). We aimed to determine the stoichiometry of TBC1D4 phosphorylation by quantitative phosphopeptide mapping of selected phosphosites by mass spectrometry. Briefly, AKT-phosphorylated TBC1D4 was separated by SDS-PAGE, digested with trypsin, and the resulting peptides were determined as described in “Experimental procedures.” As illustrated in Figure 4*B*, phosphorylation of TBC1D4 with AKT resulted in a substantial reduction in the abundance of the nonphosphorylated peptide RAHT^649^FSHPPSSSR and a concomitant increase in phospho-peptide form RAHpT^649^FSHPPSSSR, indicating that ∼80% of Thr^649^ (*i.e.*, ∼0.8 mol/mol) in TBC1D4 has been phosphorylated *in vitro*. Phosphorylated TBC1D4 was then incubated with purified [^32^P]GTP-loaded Rab10, and the amount of released [^32^P]phosphate was determined as described under “Experimental procedures.” As a result, we observed no significant difference in the GTP-hydrolysis rate of Rab10 when exposed to either nonphosphorylated or phosphorylated TBC1D4 (Fig. 4*C*). Similar results were obtained with TBC1D4 phosphorylated by AMPK (Fig. 4*C*) and phospho-TBC1D4 IMAC-purified from okadaic acid treated *Sf*9 cells or coexpression of TBC1D4 with constitutively active AKT baculovirus (Fig. S5).

### TBC1D4 interacts with 14-3-3 and IRAP in a phosphorylation-dependent manner

**Figure 5:**
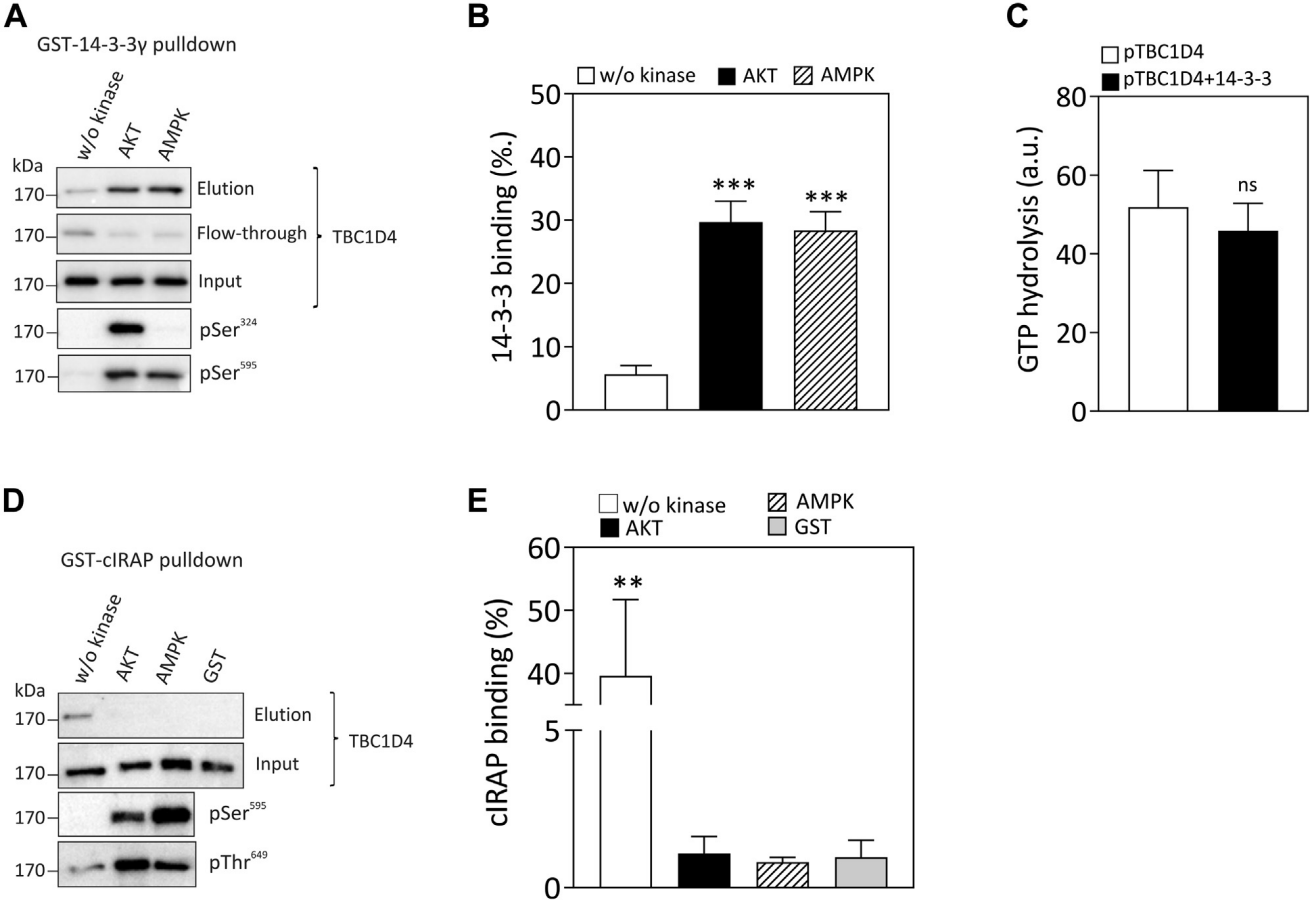
**Phosphorylation-dependent interaction of 14-3-3 and IRAP with TBC1D4 *in vitro*.***A*, GST γ14-3-3 was immobilized on GSH-Sepharose beads and incubated with 2 pmol of nonphosphorylated, AKT-phosphorylated, or AMPK-phosphorylated TBC1D4 for 1 h at 4 °C under gentle agitation as described under “Experimental procedures.” The beads were washed twice with 50 mM Tris, pH 8, and 150 mM NaCl, and bound proteins were eluted with SDS sample buffer and analyzed by western blotting using antibodies against total TBC1D1 and against the phosphosites pSer^324^ and pSer^595^, respectively. *B*, quantification of 14-3-3 binding of TBC1D4 phosphorylated by AKT or AMPK. Data represent mean values ± SEM from four independent experiments. ∗∗∗*p* < 0.0005, one-way ANOVA. *C*, 5 pmol purified phosphorylated TBC1D4 (pTBC1D4) was incubated with purified [γ-^32^P]GTP-loaded GST-Rab10 (2–6 pmol) in the absence or presence of 60 pmol 14-3-3 for 30 min at RT, and the amount of released [^32^P]phosphate was determined by scintillation counting as described under “Experimental procedures.” Data represent mean values ± SEM from four independent experiments. *D*, GST-pulldown of the cytoplasmic tail of IRAP (GST-cIRAP) and full-length TBC1D4 protein. GST-cIRAP was immobilized on GSH-Sepharose beads and incubated with 2 pmol of purified nonphosphorylated, AKT-phosphorylated, or AMPK-phosphorylated TBC1D4 purified from *Sf*9 cells for 1 h at 4 °C as described under “Experimental procedures.” Immobilized GST was used as a negative control. Eluted samples were separated by SDS-PAGE and analyzed by western blotting using TBC1D4 antibodies and phospho-TBC1D4 antibodies against Ser^595^ and Thr^649^. *E*, quantification of GST-cIRAP binding of TBC1D4 phosphorylated by AKT and AMPK. Data represent mean values ± SEM from three independent experiments. AKT-phosphorylated and AMPK-phosphorylated *versus* nonphosphorylated TBC1D4 protein ∗∗*p* < 0.0087, ns, not significant, one-way ANOVA.

Phosphorylation of TBC1D4 in response to insulin stimulation or muscle contraction has been shown to increase the binding of 14-3-3 proteins to the RabGAP (8, 21, 22). In particular, phosphorylation of Ser^348^ and Thr^649^ by AKT and AMPK was demonstrated to enhance 14-3-3 binding to TBC1D4 (8, 9). To investigate phosphorylation-dependent 14-3-3 binding to purified full-length TBC1D4 *in vitro*, GST-14-3-3γ was immobilized on GSH-Sepharose beads, incubated with nonphosphorylated TBC1D4 and AKT-and AMPK-phosphorylated TBC1D4 as described in “Experimental procedures.” As shown in Figure 5*A*, phosphorylation of Ser^324^ was specific for AKT, whereas Ser^595^ was phosphorylated by both AKT and AMPK. Binding of 14-3-3 to TBC1D4 protein was markedly increased after phosphorylation by either AKT or AMPK (Fig. 5*B*). However, the amount of 14-3-3/TBC1D4 complexes recovered was similar after phosphorylation by AKT or AMPK. We then sought to address whether phosphorylation-induced 14-3-3 binding alters TBC1D4 GAP activity. TBC1D4 was coexpressed with constitutively active AKT2 kinase and purified. As shown in Figure 5*C*, the GAP activity of phosphorylated TBC1D4 toward Rab10 was not affected by the presence of 12 M excess of 14-3-3.

The cytoplasmic tail of the GLUT4-vesicle resident protein, IRAP (insulin-regulated aminopeptidase), has been shown to interact with TBC1D4 in an insulin-dependent manner (23). To explore the impact of AKT- and AMPK-dependent phosphorylation of TBC1D4 on the interaction with IRAP, we conducted GST-pulldown assays using the immobilized 110 aa cytoplasmic tail of IRAP, GST-cIRAP and purified full-length TBC1D4 that was either nonphosphorylated or phosphorylated with AKT or AMPK (Fig. 5*D*). As shown in Figure 5*E*, nonphosphorylated TBC1D4 displayed specific binding to cIRAP, which was completely abolished after phosphorylation with either of the kinases.

### AKT- and AMPK-dependent phosphorylation interdependency of TBC1D4

**Figure 6:**
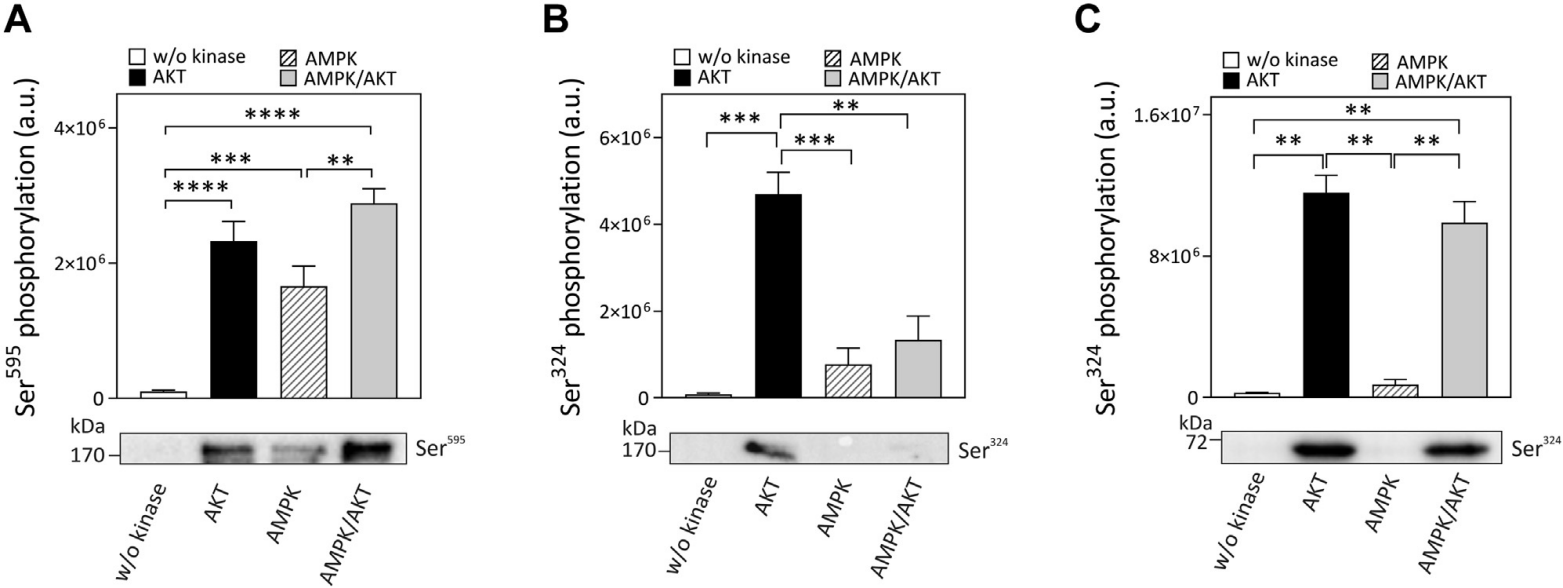
**Sequential phosphorylation of TBC1D4 by AMPK and AKT *in vitro*.** Purified TBC1D4 was phosphorylated first with purified AMPK for 5 min at RT subsequently and then with purified AKT for another 5 min. Phosphorylation was confirmed by western blotting using phosphosite-specific antibodies against pSer^324^ and pSer^595^. In parallel, single kinase assays were performed as a control for phosphorylation by each kinase. *A*, data represent mean values ± SEM from six independent experiments (∗∗∗∗*p* < 0.0001, ∗∗∗*p* = 0.0002, ∗∗*p* = 0.0067, one-way ANOVA). *B*, data represent mean values ± SEM from three independent experiments (∗∗∗*p* < 0.0007, ∗∗*p* = 0.002, one-way ANOVA). *C*, purified TBC1D4 GST-PTB2 was phosphorylated first with purified AMPK and then with purified AKT. Phosphorylation was confirmed by western blotting using phosphosite-specific antibody against pSer^324^. Data represent mean values ± SEM from two independent experiments. ∗∗*p* < 0.0041, one-way ANOVA.

Previous studies demonstrated that prior exercise enhances skeletal muscle insulin sensitivity (11, 13, 24, 25) *via* increasing the recruitment of GLUT4 transporters to the plasma membrane (26), possibly by an AMPK-mediated pathway (13). To study potential interactions at the level of specific phosphosites, we conducted sequential phosphorylation of TBC1D4 *in vitro* by adding AM*P*K first for 5 min followed by adding of AKT for another 5 min and quantification of phosphosites by immunoblotting. Ser^595^ is a preferred site for AMPK (K_M_ 10.5 μM) and comparably a minor target for AKT (K_M_ ∼70 μM; Fig. 3*C*). Conversely, Ser^324^ is a preferential target for AKT (K_M_ 6 μM) but a much poorer substrate for AMPK (∼100 μM; Fig. 3*A*). Prior AMPK phosphorylation enhanced Ser^595^ phosphorylation by AKT in an additive manner (Fig. 6*A*). However, prephosphorylation of full-length TBC1D4 with AMPK markedly inhibited subsequent phosphorylation of Ser^324^ by AKT (Fig. 6*B*). In TBC1D4, the AKT target site Ser^324^ is flanked by other potential Ser/Thr residues (Ser^320^, Ser^323^, and Thr^326^) that, if phosphorylated, could alter its phosphorylation efficiency. However, using mass spectrometry, we failed to identify corresponding tryptic phosphopeptides that would indicate phosphorylation of any of these residues in addition to Ser^324^ (data not shown). We therefore investigated a possible interaction of phosphosites by using GST fusion proteins of the second PTB domain of TBC1D4 (PTB2) as substrate. Interestingly, inhibition of Ser^324^ phosphorylation by AKT after prephosphorylation with AMPK was not observed when purified GST-PTB2 of TBC1D4 was used as a substrate (Fig. 6*C*).

## Discussion

The 160 kDa RabGAP TBC1D4 plays a critical role in insulin- and contraction-mediated GLUT4 translocation (27, 28). In the present study, we have for the first time characterized the recombinant purified enzyme with regard to structure, function, and regulation through phosphorylation by AKT and AMPK *in vitro*. Our results demonstrate that TBC1D4’s GAP activity is independent of its phosphorylation state but may be regulated through AKT/AMPK-dependent targeting to GLUT4 vesicles instead.

The RabGAP activity of TBC1D4 has been characterized by use of truncated GST fusion proteins (4, 17) as previous attempts for purification of recombinant full-length protein from mammalian cells were not successful. In this study, we have expressed and purified full-length TBC1D4 from insect cells using the baculovirus system. SEC and co-immunoprecipitation experiments indicate that full-length TBC1D4 forms oligomers of ∼600 kDa. Co-immunoprecipitations with HA- and FLAG-tagged TBC1D4 constructs show that both N-terminal PTB domains together with the ∼130 amino-acid-spanning C-terminal tail are responsible for TBC1D4 oligomerization. This is in agreement with the previous studies that indicated possible protein–protein interactions through the C-terminal region (29) and the PTB domains (21) of TBC1D4. Interestingly, mapping of tryptic sites within TBC1D4 using partial digests indicates that the regions flanking two phosphorylation targets in TBC1D4, Ser^348^ and Ser^577^, where the former is presumably involved in 14-3-3 binding (8, 9), are highly susceptible to proteolysis and thus likely more prominently exposed to the solvent than other regions of the protein. Further studies are required to address possible functional consequences of TBC1D4’s oligomeric structure.

Previous studies have investigated phosphorylation of TBC1D4 in several types of cells and tissues in response to various stimuli *in vivo*, thereby identifying up to 16 Ser/Thr phosphorylation sites that may link TBC1D4 to insulin and contraction-regulated kinases (3, 8, 11, 14, 18, 19, 20, 30, 31, 32, 33, 34, 35). Phosphorylation of eight of those sites (Ser^324^, Ser^348^, Ser^577^, Ser^595^, Thr^649^, Ser^673^, Ser^711^, and Ser^758^) has been detected in at least two independent studies (3, 8, 11, 14, 18, 19, 30, 31, 32, 34, 35). However, due to the high number of phosphorylation sites in TBC1D4, unambiguous assignment of upstream kinases and quantitative comparison of the kinetics for the different targets have been difficult. In this study, we identified 19 phosphorylation sites in TBC1D4, among those 12 have been previously reported to respond to stimulation with insulin/AICAR and/or contraction (Table 1). Using purified kinases with stable isotope-labeled [γ-^18^O_4_]ATP followed by high-resolution mass spectrometry, we mapped seven phosphorylation sites in TBC1D4 *in vitro* as primary targets for AKT (Ser^324^, Thr^649^) and AMPK (Ser^348^, Ser^577^, Ser^595^, Ser^711^, Ser^764^). Michaelis–Menten kinetics identified Ser^324^ (K_M_ ∼6 μM) and Thr^649.^ (K_M_ ∼25 μM) as preferential sites for AKT, whereas Ser^595^ (K_M_ 10 μM) and Ser^711^ (K_M_ ∼79 μM) were found to be preferred targets for AMPK. The K_M_ values of TBC1D4 sites for AKT and AMPK(α1β1γ1) are consistent with those reported for synthetic peptide substrates such as crosstide (∼16 μM) and SAMS peptide (∼27 μM), derived from AKT/AMPK phosphosites in glycogen synthase kinase-3 (GSK-3) and acetyl-CoA carboxylase (ACC), respectively (36, 37). Moreover, the AMPK preferences for the TBC1D4 sites Ser^348^, Ser^595^, and Ser^711^ are in line with *in vivo* studies of contraction/exercise-associated phosphorylation of TBC1D4 in skeletal muscle (18).

A previous study identified Ser^761^ and Ser^764^ phosphorylated in mouse skeletal muscle in response to AICAR stimulation but not contraction (20). We found Ser^761^ prephosphorylated in purified TBC1D4; however, after dephosphorylation only Ser^764^ was phosphorylated by AMPK. Similarly, Ser^673^ (equivalent to human Ser^666^), a reported target for IGF-1 and insulin (8, 35), was also found only in prephosphorylated and purified TBC1D4 while not undergoing phosphorylation by AKT *in vitro*. Ser^758^ (equivalent to human Ser^751^) was shown to be phosphorylated in response to insulin (3, 19) and exercise (11, 18), but we failed to detect this phosphorylation site.

Inconsistent results have been reported for phosphorylation of Thr^649^ in the acute response to muscle contraction, which is associated with activation of AMPK. Some studies reported either increased (18, 38), decreased (39), or unchanged phosphorylation of Thr^649^ (40), which is embedded in a canonical AKT motif (RxRxxS/T-Hyd), similar to Ser^324^. While different types of skeletal muscle, intervention protocols, and time points were analyzed in these studies, our data clearly demonstrate the preference of AKT for Thr^649^ as indicated by a 20-times lower K_M_ value compared with AMPK. In line, a knock-in mouse carrying the T649A mutation had normal glucose transport in skeletal muscle in response to contraction/AICAR but reduced response to insulin stimulation (39).

Previous studies have characterized the RabGAP activity of TBC1D4 using C-terminal–truncated fragments of the RabGAP and identified Rab8, Rab10, and Rab14 as the likely most relevant targets in regulating GLUT4 translocation. Our study revealed that the full-length protein displayed similar substrate specificity but a markedly higher specific GAP activity toward Rab10 compared with the truncated GAP domain. This could indicate that other regions than the GAP domain may participate in substrate recognition of TBC1D4 and/or that oligomerization may contribute to the higher catalytic activity of the full-length protein. Of note, the closely related TBC1D1 also exhibited substantially higher activity as full-length protein compared with the respective GAP domain (7).

Phosphorylation of TBC1D4 *in vivo* has been associated with reduced activity of the RabGAP and mutational analysis has demonstrated that Thr^649^ is particularly important as overexpression of the T649A mutation in 3T3-L1 adipocytes reduced insulin-induced GLUT4 translocation by 50% (3). Interestingly, mutation of the contraction-responsive AMPK site Ser^595^ to alanine also had an inhibitory effect on insulin-stimulated GLUT4 translocation in adipocytes albeit to a lesser degree (3, 18). Phosphorylation of TBC1D4 by AKT *in vitro* led to an almost stoichiometric phosphorylation of Thr^649^ as indicated by the conversion of the respective tryptic peptide from the dephospho- to the phospho-form. However, the GAP activity of purified TBC1D4 toward Rab10 was not significantly altered after phosphorylation with AKT, AMPK *in vitro*, or phosphorylation *in vivo* induced by cotransfection with AKT baculovirus or treatment of *Sf*9 cells with okadaic acid prior purification. Moreover, addition of excess 14-3-3 protein to phosphorylated TBC1D4 did not alter its *in vitro* GAP activity. Thus, while we cannot rule out a minor impact of phosphorylation on TBC1D4 RabGAP activity, these data are in line with our findings from the related TBC1D1 indicating that phosphorylation does not have a substantial effect on the RabGAP activity of the protein (7).

Previous studies suggest that phosphorylation regulates interaction with candidate vesicular partner proteins and/or recruitment to the GLUT4 vesicle. Our data show that phosphorylation significantly increases 14-3-3 binding to TBC1D4. A previous study showed that constitutively active binding of 14-3-3 to TBC1D4 restores the inhibitory effect of a mutant TBC1D4 lacking the four phosphorylation sites (4P mutant) in 3-T3-L1 adipocytes (22). Importantly, not all the phosphorylation sites contribute to the interaction with 14-3-3 proteins and instead only two phosphorylation sites, Ser^348^ and Thr^649^, in TBC1D4 are shown to be responsible for 14-3-3 binding (8) and subsequently for successful GLUT4 translocation (22, 41, 42). Employing limited proteolytic approach, we found that the regions close to these sites might be located outside of TBC1D4 conformation.

The insulin-regulated aminopeptidase IRAP is expressed in skeletal muscle and adipose cells and highly colocalized with GLUT4 as it constitutes a resident membrane protein of GLUT4 storage vesicles (43). Our data demonstrate that the cytoplasmic tail of IRAP binds to TBC1D4 and phosphorylation disrupts this interaction. These results are consistent with a mechanism where insulin and presumably contraction regulate access of the RabGAP to target Rab GTPases associated with GLUT4 vesicles rather than modulating the catalytic GAP activity (7, 41, 44). While this mechanism has also been proposed for the regulation of TBC1D1 (7), phosphorylation-dependent interactions of TBC1D4 and IRAP have been demonstrated also in other studies (23, 45).

Insulin and exercise signals appear to converge and may integrate at the level of TBC1D4. Prior AMPK-dependent phosphorylation of Ser^711^ induced a subsequent increase in TBC1D4 Thr^649^ phosphorylation (13). Conversely, while insulin stimulation increased Thr^649^ phosphorylation, mutation of Ser^711^ to alanine significantly decreased Thr^649^ phosphorylation, showing interdependency of the phosphorylation sites within TBC1D4 (14). In our study, we demonstrate that prior AMPK phosphorylation of TBC1D4 enhanced phosphorylation of Ser^595^ by AKT *in vitro*, which is in line with the insulin-sensitizing effect of exercise *in vivo* (46, 47). Moreover, phosphorylation of Ser^324^ by AKT was abolished after prephosphorylation of full-length TBC1D4 by AMPK. In line with our data, reduced insulin-stimulated phosphorylation of Ser^324^ in skeletal muscle was also observed after prior injection of the AMPK activator AICAR into mice (14). Our finding that no Ser/Thr residues in the vicinity of Ser^324^ were found to be phosphorylated by AMPK and phosphorylation of Ser^324^ by AKT was not affected by AMPK when the second PTB domain of TBC1D4 was used as substrate suggests more remote interactions of phosphosites within full-length TBC1D4. As the biological function of the phosphorylation of TBC1D4 remains to be clarified, further studies are required to delineate the structural context of the different phosphosites and their possible impact on effectors of TBC1D4.

## Experimental procedures

### DNA constructs

The long mouse isoform of murine *Tbc1d4* (1298 amino acids; XP_006518825.1) was cloned into the baculovirus expression vector pAcGS2-6xHis (BD Bioscience) and triple FLAG-pcDNA3.1. GST-cIRAP (Met^1^ - Thr^110^) and a baculovirus for a GST-tagged Rab10 were generated as described (7). Cloning of the GST-GAP domain of TBC1D4 and GST-AKT2ΔPH was as conducted previously (7, 17). The long isoform of *Tbc1d4* (XP_006518825.1) was cloned into the pcDNA3.1-3xFLAG and pcDNA3.1-HA. HA-tagged proteins harboring different domains of TBC1D4 (C1: Met^1^-Leu^478^; C2…C5; Fig. 1*A*) were generated by cloning respective PCR fragments into pcDNA3.1-HA. The second PTB domain of *Tbc1d4* GST-PTB2; Ser^189^ - Leu^478^ was cloned into pGEX-4T.1. All constructs were confirmed by DNA sequencing.

### Animals

Mice were kept in accordance with the NIH guidelines for the care and use of laboratory animals, and all experiments were approved by the Ethics Committee of the State Ministry of Agriculture, Nutrition, and Forestry (State of North Rhine-Westphalia, Germany). Three to six C57BL/6J mice per cage were housed at 22 °C and a 12-h light–dark cycle with *ad libitum* access to food (standard diet; Ssniff) and water. Twelve-week-old mice were injected intraperitoneally with either 1 IU/kg Insulin, 250 mg/kg AICAR, or 0.9% sterile saline as control and sacrificed by cervical dislocation after 60 min of stimulation. Tissues were removed and immediately frozen in liquid nitrogen.

### Antibodies

TBC1D4 and phospho-TBC1D4 Ser^318^, Ser^588^, and Thr^642^ (equivalent to mouse Ser^324^, Ser^595^, Thr^649^) antibodies were obtained from Cell Signaling Technology. Antibodies against FLAG and HA were from Sigma-Aldrich.

### Protein expression and purification

*Sf*9 Grace supplemented and serum-free (Sf-900TM II SFM) media were obtained from Life Technologies. *Sf*9 cells (2.5 × 10^6^ cells/ml) were infected with recombinant baculovirus at a multiplicity of infection (MOI) of 10, harvested 72 h postinfection, and lysed by mild sonication at 4 °C in lysis buffer (50 mM Hepes, pH 8, 30 mM imidazole, 0.25 M sucrose, 5 mM β-mercaptoethanol, and EDTA-free protease inhibitor tablets (Roche Diagnostics). Coexpression of the His-tagged TBC1D4 and recombinant human constitutively active AKT2 (GST-AKT2ΔPH) was performed with AKT2 recombinant baculovirus at MOI of 7 and TBC1D4 recombinant baculovirus at MOI of about 3 to 5. The phosphatase inhibitor okadaic acid (OA) was purchased from Abcam and used as a concentration of 50 nM for 30 min before harvesting the *Sf*9 cells expressing TBC1D4. Recombinant His-tagged proteins were purified by IMAC using Ni-NTA resin (Qiagen), eluted with 100 mM imidazole, 150 mM NaCl, EDTA-free protease inhibitor, pH 8, and then dialyzed against 50 mM Tris-HCl, pH 7.4. Only freshly prepared TBC1D4 protein was used in our study. GST-γ14-3-3, GST-PTB2, and GST-TBC1D4 GAP domain were expressed in *E. coli* BL21-CodonPlus (DE3)-RIL and purified by GSH affinity chromatography (Cytiva) as described (17). GST-Rab10 and constitutively active GST-AKT2ΔPH (T309E, S474D) were expressed *via* the baculovirus system and purified as described (7, 36).

### Size-exclusion chromatography

Proteins were separated by fast protein LC (FPLC) with an ENrich SEC650 column (total volume V_t_: 24 ml; Bio-Rad) in 50 mM Tris-HCl pH 7.5 at a flow rate of 1 ml/min. Standards used were blue dextrane, 2,000,000 Da for void volume (V_0_), bovine thyroglobulin, 670,000 Da; bovine γ-globulin, 158,000 Da; chicken ovalbumin, 44,000 Da; horse myoglobin, 17,000 Da; and vitamin B12, 1350 Da. Elution volumes (V_e_) were recorded to determine partition coefficients K*av* = (V_e_ − V_0_)/(V_t_ − V_0_), and the apparent M_r_ of affinity-purified TBC1D4 was calculated by regression analysis of K*av* against log M_r_ of the standards.

### RabGAP assays

GST-Rab 10 bound to GSH-Sepharose was loaded with [γ-^32^P]GTP (Hartmann Analytic) in a buffer containing 50 mM Tris-HCl, pH 8, 2.5 mM DTT and, 5 mM MgCl_2_. The matrix was washed with the same buffer to remove unbound GTP, and [γ-^32^P]GTP bound Rab 10 was eluted in 20 mM Tris-HCl, pH 7.5, 1 mM DTT, 10 mM reduced GSH, 2.5 mM MgCl_2_ and then incubated with GST-full-length or TBC1D4 GAP domain, respectively, at room temperature (RT) for 30 min. The reaction was stopped with 0.5 M EDTA, radioactive [^32^P]phosphate was separated by filtration through activated charcoal and measured by scintillation counting as described (17). Unless indicated otherwise, [^32^P]phosphate produced was normalized to the amount of radioactivity of [γ-^32^P]GTP-bound Rab proteins.

### Phosphorylation reactions

Recombinant full-length TBC1D4 was purified by IMAC using Ni-NTA resins and used as a substrate in kinase assays. Constitutively active AKT2 was expressed as a GST fusion protein in *Sf*9 cells and purified as described previously (36), and purified human AMPK(α1β1γ1) was obtained from Life Technologies. Phosphorylation reactions were carried out at RT for indicated times in the presence of 4 pmol AKT2 or AMPK, 40 mM Tris-HCl pH 7.4, 8 mM MgCl_2_, 200 μM AMP, 2 mM ATP (or [γ-^18^O_4_]ATP), and 0.4 mM dithiothreitol (DTT) in a volume of 200 μl as described (7), and substrate concentrations are indicated in the figure legends. For dephosphorylation of TBC1D4, the protein was incubated with lambda protein phosphatase (New England Biolabs), 50 mM HEPES, 1 mM MnCl_2_, 100 mM NaCl, 0.01% Brij 35, and 2 mM DDT, for 30 min at RT. Phosphatase activity was stopped by adding 10 mM sodium orthovanadate and 50 mM sodium fluoride (New England Biolabs).

### Analysis of TBC1D4 phosphorylation

Mapping of phosphosites in TBC1D4 was conducted by mass spectrometry. Briefly, kinase reactions were separated by SDS-PAGE. Coomassie-stained protein bands corresponding to TBC1D4 were excised and subjected to in-gel protein digestion as described previously (48) with minor adaptions as follows: Reduction with DTT was done at 50 °C for 15 min and each gel slice was digested with 200 ng LysC/Trypsin Mix (Promega). Lyophilized peptides were reconstituted in 1% TFA (v/v) and separated by liquid chromatography (Ultimate 3000, Thermo Fisher Scientific) using an EASYspray ion source equipped to an Orbitrap Fusion Lumos mass spectrometer (Thermo Fisher Scientific). Peptides were trapped and desalted on an Acclaim PepMap C18-LC-column (2 cm length; 164535 ThermoFisher Scientific) and subsequently separated *via* 1 and 2 h gradients on 50 cm C18-LC columns EASY-Spray (ES803A, ThermoFisher Scientific) or μPAC (552503518050, Pharmafluidics). Mass spectra were acquired in data-dependent acquisition mode and HCD as well as ETD fragmentation was used (see Supporting information).

Mass spectrometry raw files were analyzed with Proteome Discoverer 2.3/2.4 software (Thermo Fisher Scientific). HTsequest search was done against protein FASTA files from *Mus musculus* (SwissProt, TaxID = 10090, version 2017-10-25, 25,097 sequences and 2020-04, 25,264 sequences), a general contaminants list (245 sequences), and the His-tagged TBC1D4 sequence, with 10 ppm precursor mass and 0.02 Da fragment mass tolerance. Enzyme was set to trypsin with maximum two missed cleavage sites allowed. Carbamidomethylation of cysteine was set as fixed modification. N-terminal acetylation, methionine oxidation as well as phosphorylation (+79.966 Da) or “heavy” phosphorylation (+85.966 Da, transfer of phosphoryl group from [γ-^18^O_4_]ATP) of serine, threonine, or tyrosine were allowed as variable modifications. The fixed-value PSM Validator (max delta Cn: 0.05, minimal XCorr score for charge state: 2+ ≥1.9, 3+ ≥2.3, from 4+ ≥2.6) and the ptmRS-node included in Proteome Discoverer were applied. FDR was set to 0.01 (strict). Only high confident peptides and if modified with a site probability of >75% were annotated to proteins within the result file.

To estimate the phosphorylation stoichiometry of TBC1D4, we determined tryptic peptides and phosphopeptides comprising the major AKT2 phosphosite Thr^649^, (core sequence AHT^649^FSHPPSSSR) before and after phosphorylation of 2 to 3 pmol TBC1D4 using purified AKT2 at an equimolar ratio for 5 to 10 min. Abundance of nonphosphorylated and phosphorylated peptides was determined by integrating the fractional abundances of all corresponding peptide species related to AHT^649^FSHPPSSSR and AHpT^649^FSHPPSSSR. For kinetic analyses, phosphorylation of Ser^324^, Ser^595^, Thr^649^, and Ser^711^ was determined by immunoblotting using phosphosite-specific antibodies and quantification by ECL (PerkinElmer) and data were used for nonlinear curve fitting to Michaelis–Menten kinetics to estimate apparent values for K_M_ and V_max_.

### TBC1D4 co-immunoprecipitation and pulldown assays

Magnetic beads preconjugated with anti-HA monoclonal antibodies (μMACS HA-beads, Miltenyi Biotec GmbH) were used as previously described (7). Briefly, HEK293 cells coexpressing HA-*Tbc1d4* constructs and 3xFLAG-*Tbc1d4* were lysed using lysis buffer containing 150 mM NaCl, 1% Triton X-100, and 50 mM Tris-HCl, pH 8. Cleared cell lysates containing HA-tagged TBC1D4 were labeled with anti-HA magnetic beads and incubated for 30 min at 4 °C. Subsequently beads were washed with 50 mM Tris, pH 8, and 150 mM NaCl, and the protein complexes were eluted using SDS sample buffer.

In GST-pulldown assays, *E. coli* BL21-CodonPlus (DE3)-RIL cells expressing GST-cIRAP and GST-γ14-3-3 were lysed and the cleared lysate was incubated with 100 μl of GSH-Sepharose beads (Cytiva) for 2 h at 4 °C with mixing as described previously (7). As a negative control, the same amount of beads were incubated with GST alone. The beads were washed twice with 500 μl of a buffer containing 50 mM Tris, pH 8, and 150 mM NaCl and incubated with 2 pmol of nonphosphorylated and AKT2/AMPK-phosphorylated TBC1D4, respectively, for 1 h at 4 °C with gentle agitation. The beads were washed four times with 300 μl of the same buffer and eluted either with a buffer containing 10 mM reduced GSH (Sigma-Aldrich) or boiled in sample buffer. The eluted fractions were analyzed in parallel with TBC1D4 and phosphosite-TBC1D4 antibodies *via* western blot analysis.

### Statistical analysis

Data are expressed as mean ± S.E.M unless indicated otherwise. Nonlinear fitting to Michaelis–Menten was performed by using GraphPad Prism 9 (GraphPad Software, Inc). *p* values were calculated by one-way ANOVA (Tukey correction) or paired, two-tailed Student’s *t*-test, as indicated in the figure legends. *p* values <0.05 were considered statistically significant.

## Data availability

The mass spectrometry proteomics data have been deposited to the ProteomeXchange Consortium *via* the PRIDE (49) partner repository with the dataset identifiers PXD024621 and PXD024645.

## Supporting information

This article contains supporting information.

## Conflict of interest

The authors declare no conflicts of interest in regard to this article.
