# Supplementary material for: AKT/AMPK-mediated phosphorylation of TBC1D4 disrupts the interaction with insulin-regulated aminopeptidase
Source: J Biol Chem. 2021 Apr 16;296:100637. doi: 10.1016/j.jbc.2021.100637 (PMC8131924; doi:10.1016/j.jbc.2021.100637)
Supplement: Supplemental Figures S1–S5 and Table S1 [file mmc1.pdf]

Residue Position	Annotated Sequence	Precursor Mass [m/z]	Charge State	HTSequest Score [Xcorr]	Modifications	ptmRS: for indicated phospho site
Ser258	GGDPGDEMGVLEVESPVSPDDSLPEK	1376.5820	+2	2.56	**S15-Phospho** (79.9663 Da)<br>M8-Oxidation (15.9949 Da)	100
Ser261	GGDPGDEMGVLEVESPVSPDDSLPEKADGTVNSPR	1211.8689	+3	6.5	**S18-Phospho** (79.9663 Da)	99.9
Ser276	ADGTVNSPR	498.7099	+2	3.1	**S7-Phospho** (79.9663 Da)	100
Ser324	CSSVTGVMQK	591.7481	+2	2.32	**S3-heavyPhospho** (85.9663 Da)<br>C1-Carbamidomethyl (57.02146 Da)	100
Ser348	HASAPSHVQPSDSEKNR	483.9694	+4	2.81	**S3-heavyPhospho** (85.9663 Da)	100
Ser377	FEINLISPDTK	678.8242	+2	2.55	**S7-Phospho** (79.9663 Da)	100
Ser492	HLSSLTDNEQADIFER	978.7336	+2	3.64	**S3-Phospho** (79.9663 Da)	100
Ser577	SLTSSLENIFSR	720.3439	+2	2.25	**S5-heavyPhospho** (85.9663 Da)	99.65
Ser595	GRLGSMDSFER	447.5273	+3	2.8	**S5-heavyPhospho** (85.9663 Da)	100
Ser598	LGSMDSFER	561.2192	+2	3.13	**S6-Phospho** (79.9663 Da)	100
Thr649	AHTFSHPPSSSR	466.2053	+3	3.51	**T3-heavyPhospho** (85.9663 Da)	100
Ser673	AHGLRSPLLR	400.5511	+3	3.63	**S6-Phospho** (79.9663 Da)	100
Ser680	QSSSEQCSIVPSAR	808.3406	+2	3.61	**S3-Phospho** (79.9663 Da)<br>C7-Carbamidomethyl (57.02146 Da)	100
Ser711	ESNSSCSLPSLHTSFsAPSFTAPSFLK	991.7851	+3	2.61	**S16-heavyPhospho** (85.9663 Da)<br>C6-Carbamidomethyl (57.02146 Da)	99.28
Ser761	RTSSTCsNESLNAGGTPVTPR	758.3388	+3	5.88	**S7-Phospho** (79.9663 Da)<br>C6-Carbamidomethyl (57.02146 Da)	100
Ser764	RTSSTCSNEsLNAGGTPVTPR	1179.4944	+2	2.06	**S10-heavyPhospho** (85.9663 Da)<br>S7-Phospho (79.9663 Da)<br>C6-Carbamidomethyl (57.02146 Da)	99.06
Ser789	VASPVNK	397.6930	+2	2.91	**S3-Phospho** (79.9663 Da)	100
Ser794	VASPVNKsPSAMQQQK	890.9289	+2	4.74	**S8-Phospho** (79.9663 Da)	100
Ser817	DGLDRTELLPLSPLsPTMEEEPLIIFLSGDEDTEKVEEK	1121.5419	+4	2.72	**S15-Phospho** (79.9663 Da)<br>M18-Oxidation (15.9949 Da)	97.26

Ser258

Sequence: GGDPGDEMGVLEVESPVSPDDSLPEK, S15-Phospho (79.96633 Da), M8-Oxidation (15.99492 Da)
Charge: +2,   Monoisotopic m/z: 1376.08144 Da (-1.72 mmu/-1.25 ppm), MH+: 2751.15561 Da,
RT: 84.3465 min, Identified with: Sequest HT (v1.17); XCorr:2.56,
ptmRS: Best Site Probabilities:S15(Phospho): 100, Fragment match tolerance used for search: 0.02 Da

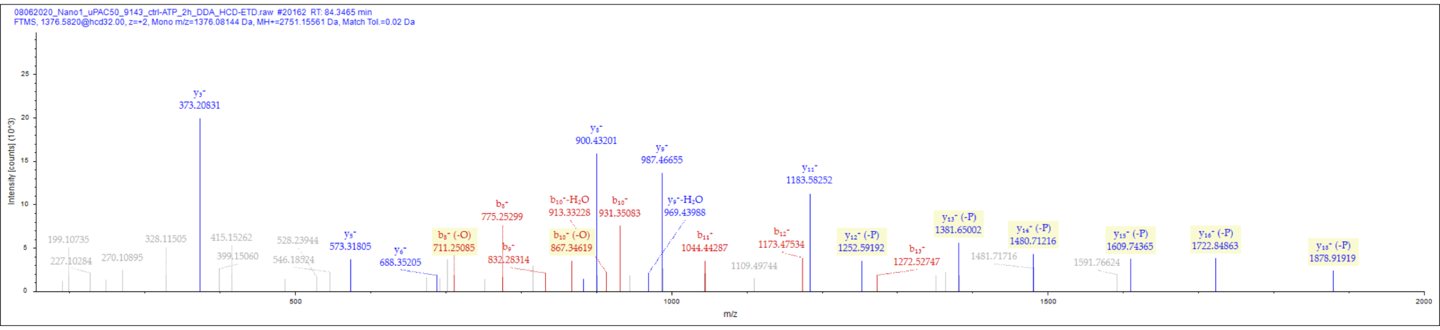

#1	b⁺	b²⁺	c⁺	c²⁺	Seq.	y⁺	y²⁺	z⁺	z+H⁺	z+H²⁺	z²⁺	z-H⁺	z-H²⁺	#2
1	58.02874	29.51801	75.05529	38.03128	G									26
2	115.05020	58.02874	132.07675	66.54201	G	2694.13758	1347.57243	2679.12668	2680.13451	1340.57089	1340.06698	2678.11886	1339.56307	25
3	230.07715	115.54221	247.10370	124.05549	D	2637.11612	1319.06170	2622.10522	2623.11304	1312.06016	1311.55625	2621.09739	1311.05233	24
4	327.12991	164.06859	344.15646	172.58187	P	2522.08917	1261.54822	2507.07827	2508.08610	1254.54669	1254.04278	2506.07045	1253.53886	23
5	384.15137	192.57933	401.17792	201.09260	G	2425.03641	1213.02184	2410.02551	2411.03334	1206.02031	1205.51639	2409.01768	1205.01248	22
6	499.17832	250.09280	516.20487	258.60607	D	2368.01495	1184.51111	2353.00405	2354.01187	1177.50957	1177.00566	2351.99622	1176.50175	21
7	628.22091	314.61409	645.24746	323.12737	E	2252.98800	1126.99764	2237.97710	2238.98493	1119.99610	1119.49219	2236.96928	1118.98828	20
8	775.25631	388.13179	792.28286	396.64507	M-Oxidation	2123.94541	1062.47634	2108.93451	2109.94234	1055.47481	1054.97089	2107.92669	1054.46698	19
9	832.27777	416.64253	849.30432	425.15580	G	1976.91001	988.95864	1961.89911	1962.90694	981.95711	981.45319	1960.89129	980.94928	18
10	931.34619	466.17673	948.37274	474.69001	V	1919.88855	960.44791	1904.87765	1905.88547	953.44637	952.94246	1903.86982	952.43855	17
11	1044.43025	522.71876	1061.45680	531.23204	L	1820.82013	910.91370	1805.80923	1806.81706	903.91217	903.40825	1804.80141	902.90434	16
12	1173.47284	587.24006	1190.49939	595.75334	E	1707.73607	854.37167	1692.72517	1693.73299	847.37014	846.86622	1691.71734	846.36231	15
13	1272.54126	636.77427	1289.56781	645.28754	V	1578.69348	789.85038	1563.68258	1564.69040	782.84884	782.34493	1562.67475	781.84101	14
14	1401.58385	701.29556	1418.61040	709.80884	E	1479.62506	740.31617	1464.61416	1465.62199	733.31463	732.81072	1463.60634	732.30681	13
15	1568.58221	784.79474	1585.60876	793.30802	S-Phospho	1350.58247	675.79487	1335.57157	1336.57939	668.79334	668.28942	1334.56374	667.78551	12
16	1665.63497	833.32113	1682.66152	841.83440	P	1183.58411	592.29569	1168.57321	1169.58104	585.29416	584.79024	1167.56539	584.28633	11
17	1764.70339	882.85533	1781.72994	891.36861	V	1086.53135	543.76931	1071.52045	1072.52827	536.76777	536.26386	1070.51262	535.75995	10
18	1851.73542	926.37135	1868.76197	934.88462	S	987.46293	494.23510	972.45203	973.45986	487.23357	486.72965	971.44421	486.22574	9
19	1948.78818	974.89773	1965.81473	983.41100	P	900.43090	450.71909	885.42000	886.42783	443.71755	443.21364	884.41218	442.70973	8
20	2063.81512	1032.41120	2080.84167	1040.92447	D	803.37814	402.19271	788.36724	789.37507	395.19117	394.68726	787.35942	394.18335	7
21	2178.84207	1089.92467	2195.86862	1098.43795	D	688.35120	344.67924	673.34030	674.34812	337.67770	337.17379	672.33247	336.66987	6
22	2265.87409	1133.44069	2282.90064	1141.95396	S	573.32425	287.16576	558.31335	559.32118	280.16423	279.66032	557.30553	279.15640	5
23	2378.95816	1189.98272	2395.98471	1198.49599	L	486.29223	243.64975	471.28133	472.28915	236.64821	236.14430	470.27350	235.64039	4
24	2476.01092	1238.50910	2493.03747	1247.02237	P	373.20816	187.10772	358.19726	359.20509	180.10618	179.60227	357.18944	179.09836	3
25	2605.05352	1303.03040	2622.08006	1311.54367	E	276.15540	138.58134	261.14450	262.15232	131.57980	131.07589	260.13667	130.57197	2
26					K	147.11280	74.06004	132.10191	133.10973	67.05850	66.55459	131.09408	66.05068	1

Ser261

Sequence: GGDPGDEMGVLEVESPVSPDDSLPEKADGTVNSPR, S18-Phospho (79.96633 Da)

Charge: +3,   Monoisotopic m/z: 1211.53455 Da (-1.83 mmu/-1.51 ppm),   MH+: 3632.58908 Da,

RT: 55.0002 min, Identified with: Sequest HT (v1.17); XCorr:6.50,

ptmRS: Best Site Probabilities:S18(Phospho): 99.99, Fragment match tolerance used for search: 0.02 Da

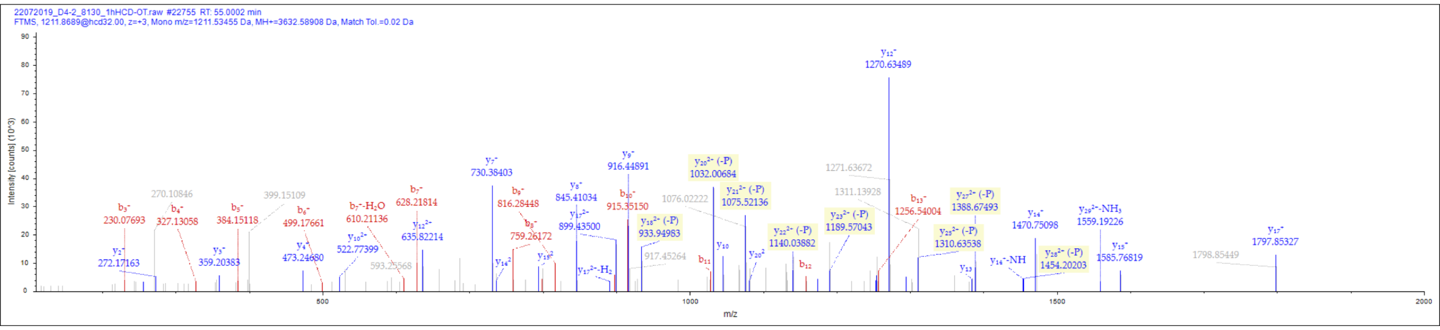

#1	b⁺	b²⁺	b³⁺	Seq.	y⁺	y²⁺	y³⁺	#2
1	58.02874	29.51801	20.01443	G				35
2	115.05020	58.02874	39.02159	G	3575.57311	1788.29019	1192.52922	34
3	230.07715	115.54221	77.36390	D	3518.55164	1759.77946	1173.52207	33
4	327.12991	164.06859	109.71482	P	3403.52470	1702.26599	1135.17975	32
5	384.15137	192.57933	128.72198	G	3306.47194	1653.73961	1102.82883	31
6	499.17832	250.09280	167.06429	D	3249.45047	1625.22888	1083.82168	30
7	628.22091	314.61409	210.07849	E	3134.42353	1567.71540	1045.47936	29
8	759.26139	380.13434	253.75865	M	3005.38094	1503.19411	1002.46516	28
9	816.28286	408.64507	272.76580	G	2874.34045	1437.67386	958.78500	27
10	915.35127	458.17927	305.78861	V	2817.31899	1409.16313	939.77785	26
11	1028.43534	514.72131	343.48330	L	2718.25058	1359.62893	906.75504	25
12	1157.47793	579.24260	386.49749	E	2605.16651	1303.08689	869.06035	24
13	1256.54634	628.77681	419.52030	V	2476.12392	1238.56560	826.04616	23
14	1385.58894	693.29811	462.53450	E	2377.05550	1189.03139	793.02335	22
15	1472.62096	736.81412	491.54517	S	2248.01291	1124.51009	750.00915	21
16	1569.67373	785.34050	523.89609	P	2160.98088	1080.99408	720.99848	20
17	1668.74214	834.87471	556.91890	V	2063.92812	1032.46770	688.64756	19
18	1835.74050	918.37389	612.58502	S-Phospho	1964.85971	982.93349	655.62475	18
19	1932.79327	966.90027	644.93594	P	1797.86135	899.43431	599.95863	17
20	2047.82021	1024.41374	683.27825	D	1700.80858	850.90793	567.60771	16
21	2162.84715	1081.92721	721.62057	D	1585.78164	793.39446	529.26540	15
22	2249.87918	1125.44323	750.63124	S	1470.75470	735.88099	490.92308	14
23	2362.96324	1181.98526	788.32593	L	1383.72267	692.36497	461.91241	13
24	2460.01601	1230.51164	820.67685	P	1270.63860	635.82294	424.21772	12
25	2589.05860	1295.03294	863.69105	E	1173.58584	587.29656	391.86680	11
26	2717.15356	1359.08042	906.38937	K	1044.54325	522.77526	348.85260	10
27	2788.19068	1394.59898	930.06841	A	916.44828	458.72778	306.15428	9
28	2903.21762	1452.11245	968.41072	D	845.41117	423.20922	282.47524	8
29	2960.23908	1480.62318	987.41788	G	730.38423	365.69575	244.13293	7
30	3061.28676	1531.14702	1021.10044	T	673.36276	337.18502	225.12577	6
31	3160.35518	1580.68123	1054.12324	V	572.31509	286.66118	191.44321	5
32	3274.39810	1637.70269	1092.13755	N	473.24667	237.12697	158.42041	4
33	3361.43013	1681.21870	1121.14823	S	359.20374	180.10551	120.40610	3
34	3458.48290	1729.74509	1153.49915	P	272.17172	136.58950	91.39542	2
35				R	175.11895	88.06311	59.04450	1

Ser276

Sequence: ADGTVNSPR, S7-Phospho (79.96633 Da)

Charge: +2,   Monoisotopic m/z: 498.70993 Da (-1.02 mmu/-2.04 ppm),   MH+: 996.41258 Da,

RT: 19.8079 min, Identified with: Sequest HT (v1.17); XCorr:3.10,

ptmRS: Best Site Probabilities:S7(Phospho): 100, Fragment match tolerance used for search: 0.02 Da

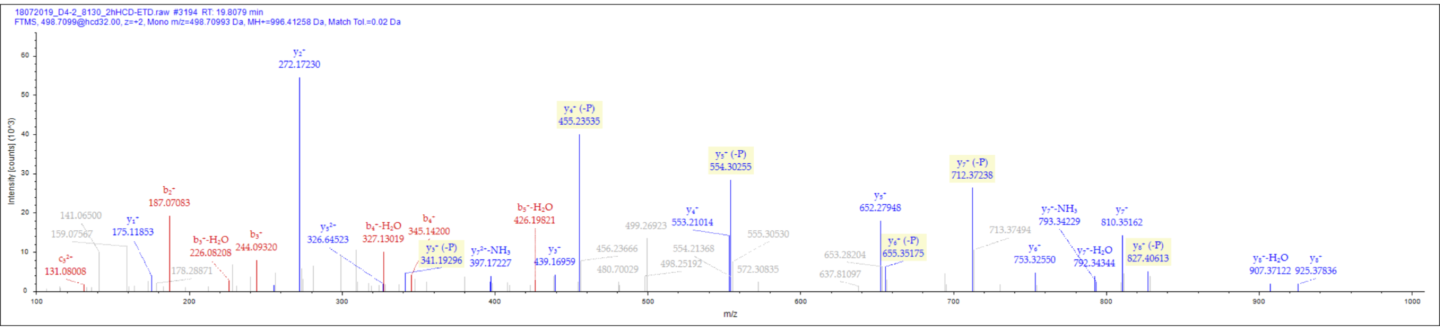

#1	b^+	b^{2+}	c^+	c^{2+}	Seq.	y^+	y^{2+}	z^+	$z+H^+$	$z+H^{2+}$	z^{2+}	$z-H^+$	$z-H^{2+}$	#2
1	72.04439	36.52583	89.07094	45.03911	A									9
2	187.07133	94.03930	204.09788	102.55258	D	925.37750	463.19239	910.36660	911.37443	456.19085	455.68694	909.35878	455.18303	8
3	244.09280	122.55004	261.11935	131.06331	G	810.35056	405.67892	795.33966	796.34748	398.67738	398.17347	794.33183	397.66956	7
4	345.14048	173.07388	362.16702	181.58715	T	753.32910	377.16819	738.31820	739.32602	370.16665	369.66274	737.31037	369.15882	6
5	444.20889	222.60808	461.23544	231.12136	V	652.28142	326.64435	637.27052	638.27834	319.64281	319.13890	636.26269	318.63498	5
6	558.25182	279.62955	575.27837	288.14282	N	553.21300	277.11014	538.20210	539.20993	270.10860	269.60469	537.19428	269.10078	4
7	725.25018	363.12873	742.27673	371.64200	S-Phospho	439.17008	220.08868	424.15918	425.16700	213.08714	212.58323	423.15135	212.07931	3
8	822.30294	411.65511	839.32949	420.16838	P	272.17172	136.58950	257.16082	258.16864	129.58796	129.08405	256.15299	128.58013	2
9					R	175.11895	88.06311	160.10805	161.11588	81.06158	80.55766	159.10023	80.05375	1

Ser324

Sequence: CSSVTGVMQK, C1-Carbamidomethyl (57.02146 Da), S3-heavyPhospho (85.96631 Da)
Charge: +2, Monoisotopic m/z: 591.74811 Da (+5.05 mmu/+8.53 ppm), MH+: 1182.48894 Da,
RT: 41.5046 min,Identified with: Sequest HT (v1.17); XCorr:2.32,
ptmRS: Best Site Probabilities:S3(heavyPhospho): 100, Fragment match tolerance used for search: 0.02 Da

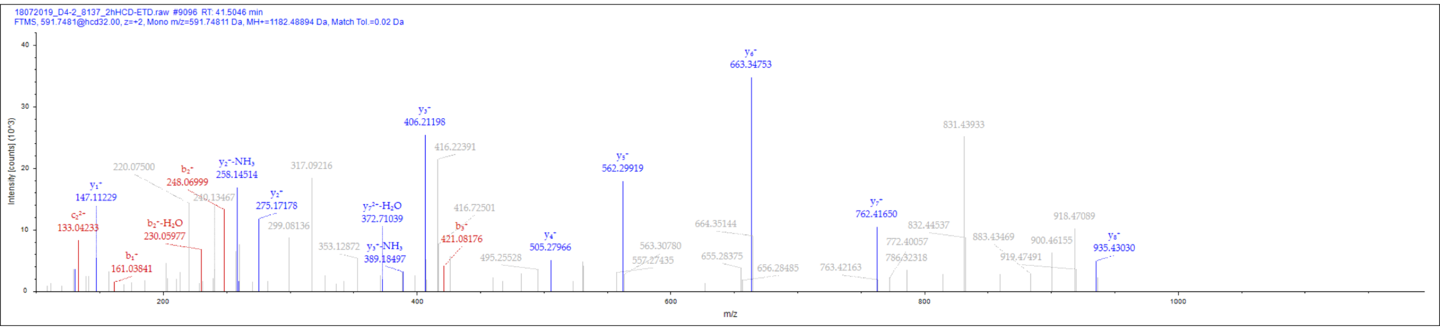

#1	b+	b2+	c+	c2+	Seq.	y+	y2+	z+	z+H+	z+H2+	z2+	z-H+	z-H2+	#2
1	161.03793	81.02260	178.06447	89.53588	C-Carbami...									10
2	248.06995	124.53862	265.09650	133.05189	S	1022.44820	511.72774	1007.43730	1008.44513	504.72620	504.22229	1006.42948	503.71838	9
3	421.06829	211.03778	438.09484	219.55106	S-heavyPh...	935.41617	468.21173	920.40528	921.41310	461.21019	460.70628	919.39745	460.20236	8
4	520.13671	260.57199	537.16325	269.08527	V	762.41784	381.71256	747.40694	748.41476	374.71102	374.20711	746.39911	373.70319	7
5	621.18438	311.09583	638.21093	319.60910	T	663.34942	332.17835	648.33852	649.34635	325.17681	324.67290	647.33070	324.16899	6
6	678.20585	339.60656	695.23240	348.11984	G	562.30174	281.65451	547.29084	548.29867	274.65297	274.14906	546.28302	273.64515	5
7	777.27426	389.14077	794.30081	397.65404	V	505.28028	253.14378	490.26938	491.27721	246.14224	245.63833	489.26156	245.13442	4
8	908.31475	454.66101	925.34130	463.17429	M	406.21187	203.60957	391.20097	392.20879	196.60803	196.10412	390.19314	195.60021	3
9	1036.37332	518.69030	1053.39987	527.20357	Q	275.17138	138.08933	260.16048	261.16831	131.08779	130.58388	259.15266	130.07997	2
10					K	147.11280	74.06004	132.10191	133.10973	67.05850	66.55459	131.09408	66.05068	1

Ser348

Sequence: HASAPSHVQPSDSEKNR, S3-heavyPhospho (85.96631 Da)

Charge: +4, Monoisotopic m/z: 483.96932 Da (+2.53 mmu/+5.22 ppm), MH+: 1932.85546 Da,

RT: 42.2567 min, Identified with: Sequest HT (v1.17); XCorr:2.81,

ptmRS: Best Site Probabilities:S3(heavyPhospho): 100,Fragment match tolerance used for search: 0.02 Da

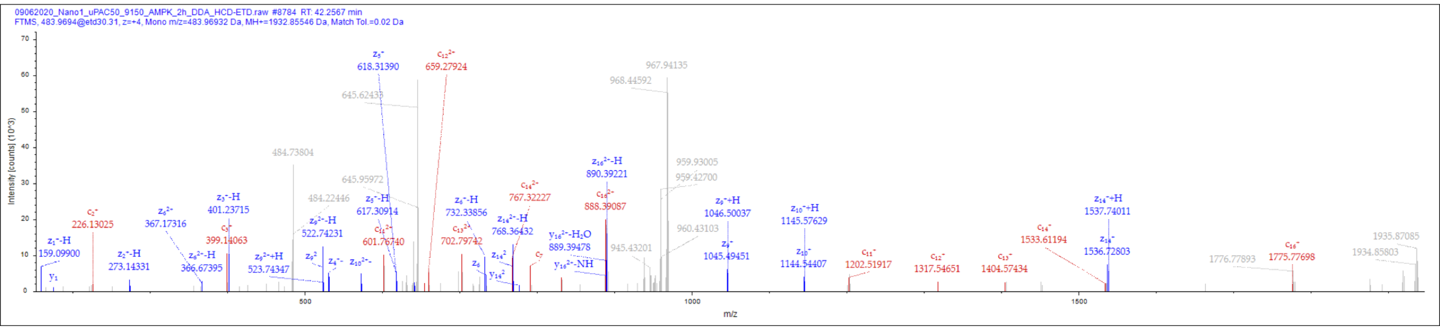

#1	b^+	b^{2+}	b^{3+}	c^+	c^{2+}	c^{3+}	Seq.	y^+	y^{2+}	y^{3+}	z^+	$z+H^+$	$z+H^{2+}$	$z+H^{3+}$	z^{2+}	z^{3+}	$z-H^+$	$z-H^{2+}$	$z-H^{3+}$	#2
1	138.06619	69.53673	46.69358	155.09274	78.05001	52.36910	H													17
2	209.10330	105.05529	70.37262	226.12985	113.56856	76.04813	A	1795.78646	898.39687	599.26700	1780.77556	1781.78339	891.39533	594.59931	890.89142	594.26337	1779.76774	890.38751	593.92743	16
3	382.10164	191.55446	128.03873	399.12819	200.06773	133.71425	S-heavyPh...	1724.74935	862.87831	575.58797	1709.73845	1710.74627	855.87677	570.92028	855.37286	570.58433	1708.73062	854.86895	570.24839	15
4	453.13875	227.07302	151.71777	470.16530	235.58629	157.39329	A	1551.75101	776.37914	517.92185	1536.74011	1537.74793	769.37761	513.25416	768.87369	512.91822	1535.73228	768.36978	512.58228	14
5	550.19152	275.59940	184.06869	567.21807	284.11267	189.74421	P	1480.71389	740.86059	494.24282	1465.70300	1466.71082	733.85905	489.57512	733.35514	489.23918	1464.69517	732.85122	488.90324	13
6	637.22355	319.11541	213.07937	654.25010	327.62869	218.75488	S	1383.66113	692.33420	461.89189	1368.65023	1369.65806	685.33267	457.22420	684.82875	456.88826	1367.64241	684.32484	456.55232	12
7	774.28246	387.64487	258.76567	791.30901	396.15814	264.44119	H	1296.62910	648.81819	432.88122	1281.61820	1282.62603	641.81665	428.21353	641.31274	427.87759	1280.61038	640.80883	427.54164	11
8	873.35087	437.17907	291.78848	890.37742	445.69235	297.46399	V	1159.57019	580.28873	387.19491	1144.55929	1145.56712	573.28720	382.52722	572.78328	382.19128	1143.55147	572.27937	381.85534	10
9	1001.40945	501.20836	334.47467	1018.43600	509.72164	340.15018	Q	1060.50178	530.75453	354.17211	1045.49088	1046.49870	523.75299	349.50442	523.24908	349.16848	1044.48305	522.74516	348.83254	9
10	1098.46221	549.73475	366.82559	1115.48876	558.24802	372.50111	P	932.44320	466.72524	311.48592	917.43230	918.44013	459.72370	306.81823	459.21979	306.48228	916.42448	458.71588	306.14634	8
11	1185.49424	593.25076	395.83626	1202.52079	601.76403	401.51178	S	835.39044	418.19886	279.13500	820.37954	821.38736	411.19732	274.46730	410.69341	274.13136	819.37171	410.18949	273.79542	7
12	1300.52118	650.76423	434.17858	1317.54773	659.27751	439.85410	D	748.35841	374.68284	250.12432	733.34751	734.35533	367.68130	245.45663	367.17739	245.12069	732.33968	366.67348	244.78475	6
13	1387.55321	694.28024	463.18926	1404.57976	702.79352	468.86477	S	633.33146	317.16937	211.78201	618.32056	619.32839	310.16783	207.11431	309.66392	206.77837	617.31274	309.16001	206.44243	5
14	1516.59581	758.80154	506.20345	1533.62236	767.31482	511.87897	E	546.29944	273.65336	182.77133	531.28854	532.29636	266.65182	178.10364	266.14791	177.76770	530.28071	265.64399	177.43175	4
15	1644.69077	822.84902	548.90177	1661.71732	831.36230	554.57729	K	417.25684	209.13206	139.75713	402.24594	403.25377	202.13052	135.08944	201.62661	134.75350	401.23812	201.12270	134.41756	3
16	1758.73370	879.87049	586.91608	1775.76025	888.38376	592.59160	N	289.16188	145.08458	97.05881	274.15098	275.15881	138.08304	92.39112	137.57913	92.05518	273.14316	137.07522	91.71924	2
17							R	175.11895	88.06311	59.04450	160.10805	161.11588	81.06158	54.37681	80.55766	54.04087	159.10023	80.05375	53.70493	1

Ser377

Sequence: FEINLISPDTK, S7-Phospho (79.96633 Da)

Charge: +2, Monoisotopic m/z: 678.82422 Da (-1.76 mmu/-2.59 ppm), MH+: 1356.64116 Da,

RT: 87.7573 min, Identified with: Sequest HT (v1.17); XCorr:2.55,

ptmRS: Best Site Probabilities:S7(Phospho): 100, Fragment match tolerance used for search: 0.02 Da

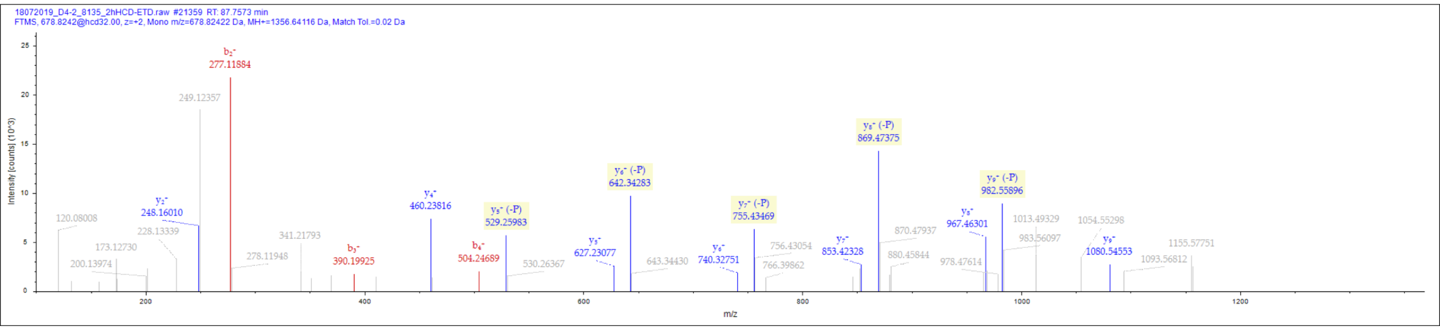

#1	b^+	b^{2+}	c^+	c^{2+}	Seq.	y^+	y^{2+}	z^+	$z{+}H^+$	$z{+}H^{2+}$	z^{2+}	$z{-}H^+$	$z{-}H^{2+}$	#2
1	148.07569	74.54148	165.10224	83.05476	F									11
2	277.11828	139.06278	294.14483	147.57605	E	1209.57626	605.29177	1194.56536	1195.57319	598.29023	597.78632	1193.55754	597.28241	10
3	390.20235	195.60481	407.22890	204.11809	I	1080.53367	540.77047	1065.52277	1066.53059	533.76894	533.26502	1064.51494	532.76111	9
4	504.24527	252.62628	521.27182	261.13955	N	967.44960	484.22844	952.43870	953.44653	477.22690	476.72299	951.43088	476.21908	8
5	617.32934	309.16831	634.35589	317.68158	L	853.40668	427.20698	838.39578	839.40360	420.20544	419.70153	837.38795	419.19761	7
6	730.41340	365.71034	747.43995	374.22361	I	740.32261	370.66494	725.31171	726.31954	363.66341	363.15950	724.30389	362.65558	6
7	897.41176	449.20952	914.43831	457.72279	S-Phospho	627.23855	314.12291	612.22765	613.23547	307.12138	306.61746	611.21982	306.11355	5
8	994.46453	497.73590	1011.49107	506.24918	P	460.24019	230.62373	445.22929	446.23712	223.62220	223.11828	444.22147	222.61437	4
9	1109.49147	555.24937	1126.51802	563.76265	D	363.18743	182.09735	348.17653	349.18435	175.09581	174.59190	347.16870	174.08799	3
10	1210.53915	605.77321	1227.56570	614.28649	T	248.16048	124.58388	233.14958	234.15741	117.58234	117.07843	232.14176	116.57452	2
11					K	147.11280	74.06004	132.10191	133.10973	67.05850	66.55459	131.09408	66.05068	1

Ser492

Sequence: HLSSLTDNEQADIFER, S3-Phospho (79.96633 Da)

Charge: +2, Monoisotopic m/z: 977.92963 Da (-1.13 mmu/-1.15 ppm), MH+: 1954.85198 Da,

RT: 74.2388 min, Identified with: Sequest HT (v1.17); XCorr:3.64,

ptmRS: Best Site Probabilities:S3(Phospho): 100, Fragment match tolerance used for search: 0.02 Da

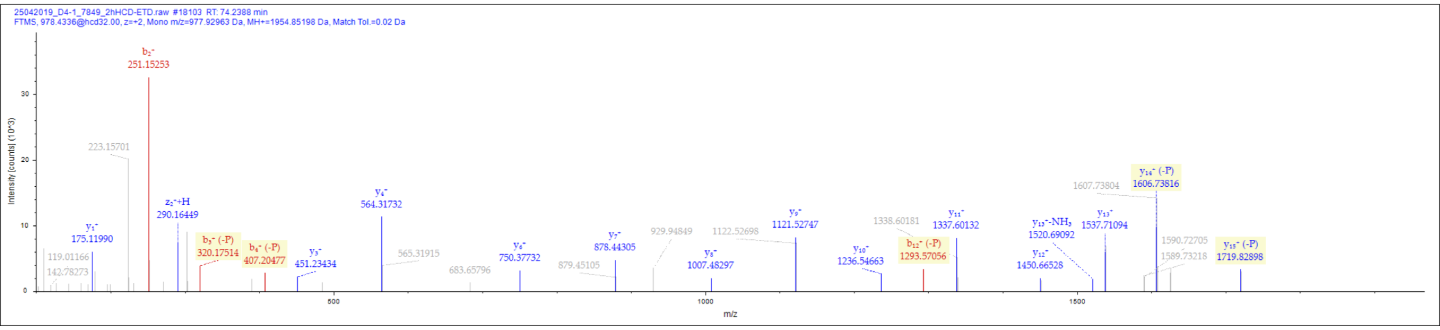

#1	b⁺	b²⁺	c⁺	c²⁺	Seq.	y⁺	y²⁺	z⁺	z+H⁺	z+H²⁺	z²⁺	z-H⁺	z-H²⁺	#2
1	138.06619	69.53673	155.09274	78.05001	H									16
2	251.15025	126.07876	268.17680	134.59204	L	1817.79531	909.40130	1802.78442	1803.79224	902.39976	901.89585	1801.77659	901.39193	15
3	418.14861	209.57794	435.17516	218.09122	S-Phospho	1704.71125	852.85926	1689.70035	1690.70818	845.85773	845.35381	1688.69253	844.84990	14
4	505.18064	253.09396	522.20719	261.60723	S	1537.71289	769.36008	1522.70199	1523.70982	762.35855	761.85463	1521.69417	761.35072	13
5	618.26470	309.63599	635.29125	318.14926	L	1450.68086	725.84407	1435.66996	1436.67779	718.84253	718.33862	1434.66214	717.83471	12
6	719.31238	360.15983	736.33893	368.67310	T	1337.59680	669.30204	1322.58590	1323.59372	662.30050	661.79659	1321.57807	661.29268	11
7	834.33933	417.67330	851.36587	426.18658	D	1236.54912	618.77820	1221.53822	1222.54605	611.77666	611.27275	1220.53040	610.76884	10
8	948.38225	474.69476	965.40880	483.20804	N	1121.52218	561.26473	1106.51128	1107.51910	554.26319	553.75928	1105.50345	553.25537	9
9	1077.42485	539.21606	1094.45139	547.72934	E	1007.47925	504.24326	992.46835	993.47618	497.24173	496.73781	991.46053	496.23390	8
10	1205.48342	603.24535	1222.50997	611.75862	Q	878.43666	439.72197	863.42576	864.43358	432.72043	432.21652	862.41793	431.71260	7
11	1276.52054	638.76391	1293.54709	647.27718	A	750.37808	375.69268	735.36718	736.37501	368.69114	368.18723	734.35936	367.68332	6
12	1391.54748	696.27738	1408.57403	704.79065	D	679.34097	340.17412	664.33007	665.33789	333.17258	332.66867	663.32224	332.16476	5
13	1504.63154	752.81941	1521.65809	761.33268	I	564.31402	282.66065	549.30312	550.31095	275.65911	275.15520	548.29530	274.65129	4
14	1651.69996	826.35362	1668.72651	834.86689	F	451.22996	226.11862	436.21906	437.22689	219.11708	218.61317	435.21124	218.10926	3
15	1780.74255	890.87491	1797.76910	899.38819	E	304.16155	152.58441	289.15065	290.15847	145.58287	145.07896	288.14282	144.57505	2
16					R	175.11895	88.06311	160.10805	161.11588	81.06158	80.55766	159.10023	80.05375	1

Ser577

Sequence: SLTSSLENIFSR, S5-heavyPhospho (85.96631 Da)

Charge: +2,   Monoisotopic m/z: 720.34387 Da (+6.65 mmu/+9.23 ppm),   MH+: 1439.68047 Da,

RT: 100.6558 min, Identified with: Sequest HT (v1.17); XCorr:2.25,

ptmRS: Best Site Probabilities:S5(heavyPhospho): 99.65, Fragment match tolerance used for search: 0.02 Da

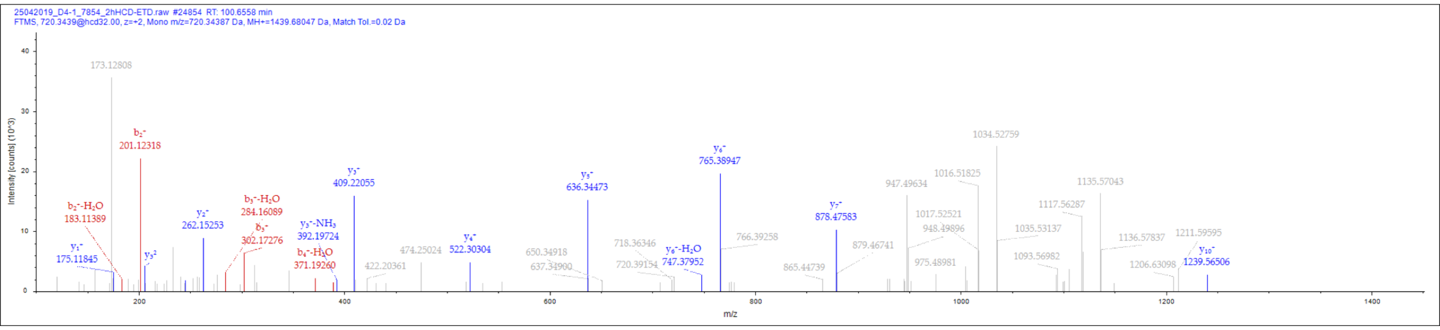

#1	b+	b2+	c+	c2+	Seq.	y+	y2+	z+	z+H+	z+H2+	z2+	z-H+	z-H2+	#2
1	88.03930	44.52329	105.06585	53.03657	S									12
2	201.12337	101.06532	218.14992	109.57860	L	1352.63515	676.82121	1337.62425	1338.63208	669.81968	669.31576	1336.61643	668.81185	11
3	302.17105	151.58916	319.19760	160.10244	T	1239.55109	620.27918	1224.54019	1225.54801	613.27765	612.77373	1223.53236	612.26982	10
4	389.20308	195.10518	406.22962	203.61845	S	1138.50341	569.75534	1123.49251	1124.50034	562.75381	562.24989	1122.48469	561.74598	9
5	562.20141	281.60435	579.22796	290.11762	S-heavyPh...	1051.47138	526.23933	1036.46048	1037.46831	519.23779	518.73388	1035.45266	518.22997	8
6	675.28548	338.14638	692.31203	346.65965	L	878.47304	439.74016	863.46214	864.46997	432.73862	432.23471	862.45432	431.73080	7
7	804.32807	402.66767	821.35462	411.18095	E	765.38898	383.19813	750.37808	751.38590	376.19659	375.69268	749.37025	375.18877	6
8	918.37100	459.68914	935.39755	468.20241	N	636.34639	318.67683	621.33549	622.34331	311.67529	311.17138	620.32766	310.66747	5
9	1031.45506	516.23117	1048.48161	524.74444	I	522.30346	261.65537	507.29256	508.30038	254.65383	254.14992	506.28473	253.64601	4
10	1178.52348	589.76538	1195.55003	598.27865	F	409.21939	205.11334	394.20850	395.21632	198.11180	197.60789	393.20067	197.10397	3
11	1265.55550	633.28139	1282.58205	641.79467	S	262.15098	131.57913	247.14008	248.14791	124.57759	124.07368	246.13226	123.56977	2
12					R	175.11895	88.06311	160.10805	161.11588	81.06158	80.55766	159.10023	80.05375	1

Ser595

Sequence: GRLGSMDSFER, S5-heavyPhospho (85.96631 Da)

Charge: +3,   Monoisotopic m/z: 447.52725 Da (+3.79 mmu/+8.46 ppm),   MH+: 1340.56720 Da,

RT: 53.8501 min, Identified with: Sequest HT (v1.17); XCorr:2.80,

ptmRS: Best Site Probabilities:S5(heavyPhospho): 100, Fragment match tolerance used for search: 0.02 Da

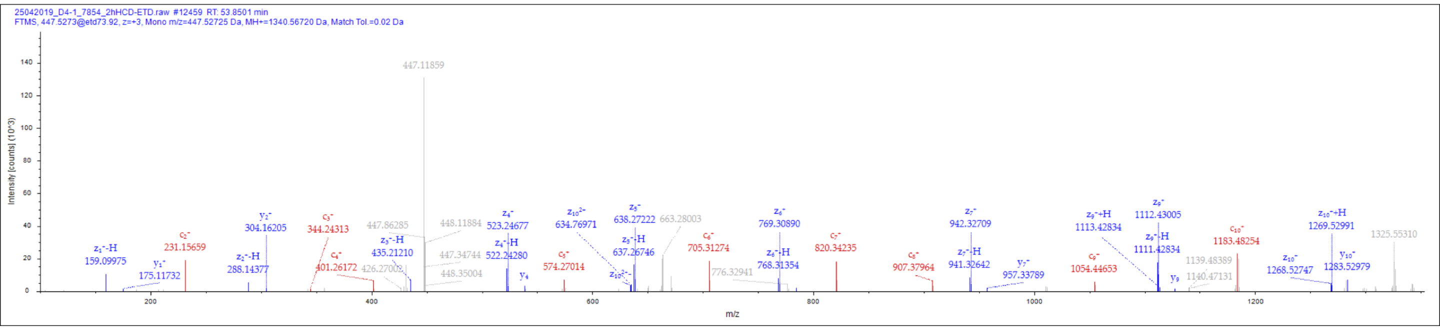

#1	b⁺	b²⁺	c⁺	c²⁺	Seq.	y⁺	y²⁺	z⁺	z+H⁺	z+H²⁺	z²⁺	z-H⁺	z-H²⁺	#2
1	58.02874	29.51801	75.05529	38.03128	G									11
2	214.12985	107.56856	231.15640	116.08184	R	1283.53439	642.27083	1268.52349	1269.53132	635.26930	634.76538	1267.51567	634.26147	10
3	327.21392	164.11060	344.24046	172.62387	L	1127.43328	564.22028	1112.42238	1113.43021	557.21874	556.71483	1111.41456	556.21092	9
4	384.23538	192.62133	401.26193	201.13460	G	1014.34922	507.67825	999.33832	1000.34614	500.67671	500.17280	998.33049	499.66888	8
5	557.23372	279.12050	574.26027	287.63377	S-heavyPh...	957.32775	479.16751	942.31685	943.32468	472.16598	471.66207	941.30903	471.15815	7
6	688.27420	344.64074	705.30075	353.15401	M	784.32942	392.66835	769.31852	770.32634	385.66681	385.16290	768.31069	384.65898	6
7	803.30114	402.15421	820.32769	410.66749	D	653.28893	327.14810	638.27803	639.28586	320.14657	319.64265	637.27021	319.13874	5
8	890.33317	445.67022	907.35972	454.18350	S	538.26199	269.63463	523.25109	524.25891	262.63310	262.12918	522.24326	261.62527	4
9	1037.40159	519.20443	1054.42814	527.71771	F	451.22996	226.11862	436.21906	437.22689	219.11708	218.61317	435.21124	218.10926	3
10	1166.44418	583.72573	1183.47073	592.23900	E	304.16155	152.58441	289.15065	290.15847	145.58287	145.07896	288.14282	144.57505	2
11					R	175.11895	88.06311	160.10805	161.11588	81.06158	80.55766	159.10023	80.05375	1

Ser598

Sequence: LGSMDSFER, S6-Phospho (79.96633 Da)

Charge: +2, Monoisotopic m/z: 561.21924 Da (-1.05 mmu/-1.87 ppm), MH+: 1121.43120 Da,

RT: 63.1517 min, Identified with: Sequest HT (v1.17); XCorr:3.13,

ptmRS: Best Site Probabilities:S6(Phospho): 100, Fragment match tolerance used for search: 0.02 Da

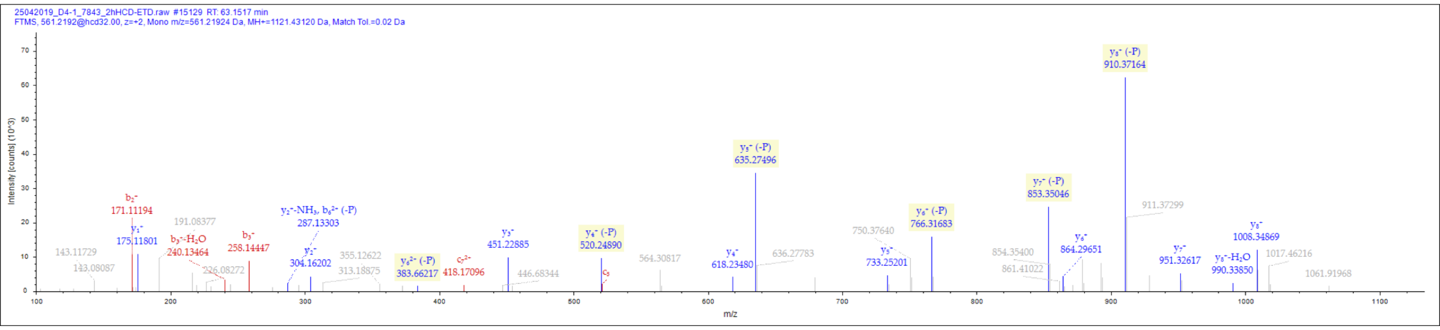

#1	b⁺	b²⁺	c⁺	c²⁺	Seq.	y⁺	y²⁺	z⁺	z+H⁺	z+H²⁺	z²⁺	z-H⁺	z-H²⁺	#2
1	114.09134	57.54931	131.11789	66.06258	L									9
2	171.11280	86.06004	188.13935	94.57331	G	1008.34924	504.67826	993.33834	994.34616	497.67672	497.17281	992.33051	496.66890	8
3	258.14483	129.57605	275.17138	138.08933	S	951.32777	476.16753	936.31688	937.32470	469.16599	468.66208	935.30905	468.15816	7
4	389.18532	195.09630	406.21187	203.60957	M	864.29575	432.65151	849.28485	850.29267	425.64997	425.14606	848.27702	424.64215	6
5	504.21226	252.60977	521.23881	261.12304	D	733.25526	367.13127	718.24436	719.25219	360.12973	359.62582	717.23654	359.12191	5
6	671.21062	336.10895	688.23717	344.62222	S-Phospho	618.22832	309.61780	603.21742	604.22524	302.61626	302.11235	602.20959	301.60844	4
7	818.27903	409.64315	835.30558	418.15643	F	451.22996	226.11862	436.21906	437.22689	219.11708	218.61317	435.21124	218.10926	3
8	947.32163	474.16445	964.34818	482.67773	E	304.16155	152.58441	289.15065	290.15847	145.58287	145.07896	288.14282	144.57505	2
9					R	175.11895	88.06311	160.10805	161.11588	81.06158	80.55766	159.10023	80.05375	1

Thr649

Sequence: AHTFSHPPSSSR, T3-heavyPhospho (85.96631 Da)

Charge: +3, Monoisotopic m/z: 466.20529 Da (+3.8 mmu/+8.15 ppm), MH+: 1396.60132 Da,

RT: 20.2793 min, Identified with: Sequest HT (v1.17); XCorr:3.51,

ptmRS: Best Site Probabilities:T3(heavyPhospho): 100, Fragment match tolerance used for search: 0.02 Da

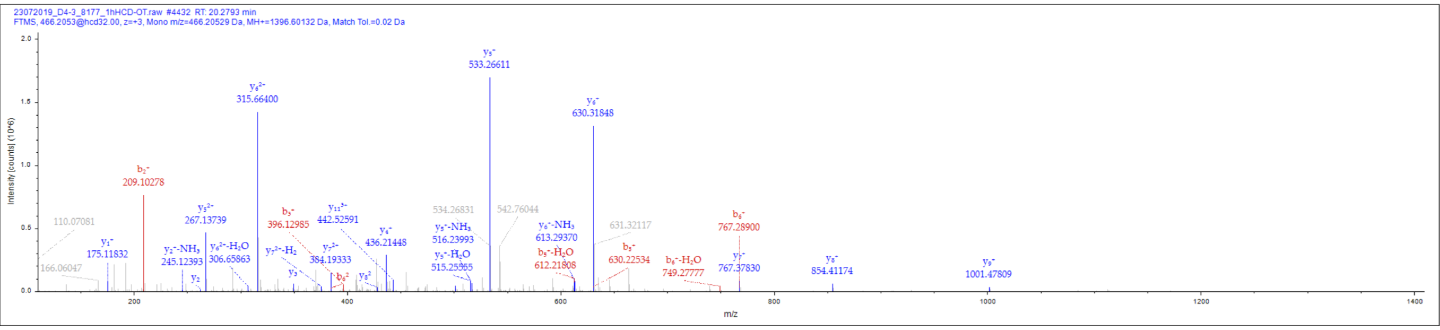

#1	b+	b2+	b3+	Seq.	y+	y2+	y3+	#2
1	72.04439	36.52583	24.68631	A				12
2	209.10330	105.05529	70.37262	H	1325.55282	663.28005	442.52246	11
3	396.11729	198.56228	132.71061	T-heavyPh...	1188.49391	594.75059	396.83615	10
4	543.18570	272.09649	181.73342	F	1001.47992	501.24360	334.49816	9
5	630.21773	315.61250	210.74410	S	854.41151	427.70939	285.47535	8
6	767.27664	384.14196	256.43040	H	767.37948	384.19338	256.46468	7
7	864.32941	432.66834	288.78132	P	630.32056	315.66392	210.77837	6
8	961.38217	481.19472	321.13224	P	533.26780	267.13754	178.42745	5
9	1048.41420	524.71074	350.14292	S	436.21504	218.61116	146.07653	4
10	1135.44623	568.22675	379.15359	S	349.18301	175.09514	117.06585	3
11	1222.47826	611.74277	408.16427	S	262.15098	131.57913	88.05518	2
12				R	175.11895	88.06311	59.04450	1

Ser673

Sequence: AHGLRSPLLR, S6-Phospho (79.96633 Da)

Charge: +3, Monoisotopic m/z: 400.55106 Da (-0.75 mmu/-1.88 ppm), MH+: 1199.63861 Da,

RT: 36.4646 min, Identified with: Sequest HT (v1.17); XCorr:3.63,

ptmRS: Best Site Probabilities:S6(Phospho): 100, Fragment match tolerance used for search: 0.02 Da

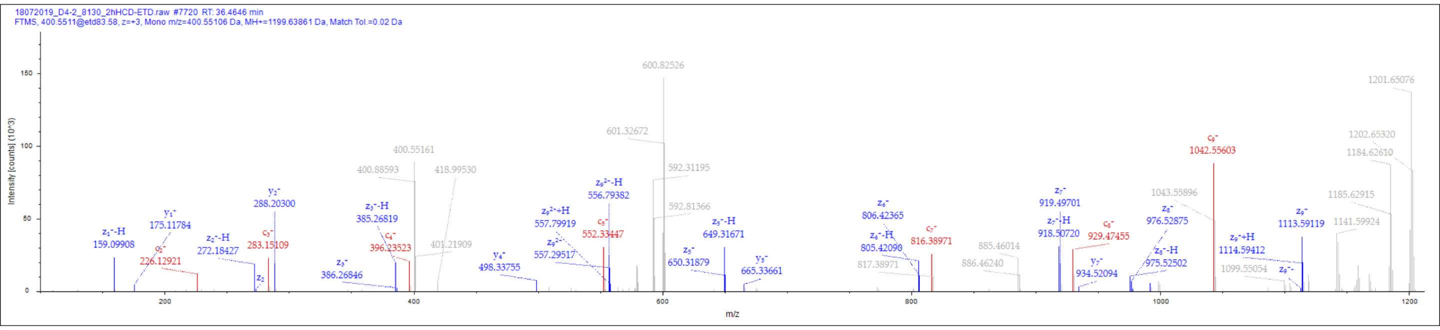

#1	b^+	b^{2+}	c^+	c^{2+}	Seq.	y^+	y^{2+}	z^+	$z+H^+$	$z+H^{2+}$	z^{2+}	$z-H^+$	$z-H^{2+}$	#2
1	72.04439	36.52583	89.07094	45.03911	A									10
2	209.10330	105.05529	226.12985	113.56856	H	1128.60375	564.80552	1113.59285	1114.60068	557.80398	557.30007	1112.58503	556.79615	9
3	266.12477	133.56602	283.15131	142.07930	G	991.54484	496.27606	976.53394	977.54177	489.27452	488.77061	975.52612	488.26670	8
4	379.20883	190.10805	396.23538	198.62133	L	934.52338	467.76533	919.51248	920.52030	460.76379	460.25988	918.50465	459.75597	7
5	535.30994	268.15861	552.33649	276.67188	R	821.43931	411.22330	806.42842	807.43624	404.22176	403.71785	805.42059	403.21393	6
6	702.30830	351.65779	719.33485	360.17106	S-Phospho	665.33820	333.17274	650.32730	651.33513	326.17120	325.66729	649.31948	325.16338	5
7	799.36106	400.18417	816.38761	408.69744	P	498.33984	249.67356	483.32894	484.33677	242.67202	242.16811	482.32112	241.66420	4
8	912.44513	456.72620	929.47168	465.23948	L	401.28708	201.14718	386.27618	387.28401	194.14564	193.64173	385.26836	193.13782	3
9	1025.52919	513.26823	1042.55574	521.78151	L	288.20302	144.60515	273.19212	274.19994	137.60361	137.09970	272.18429	136.59578	2
10					R	175.11895	88.06311	160.10805	161.11588	81.06158	80.55766	159.10023	80.05375	1

Ser680

Sequence: QSSSEQCSIVPSAR, C7-Carbamidomethyl (57.02146 Da), S3-Phospho (79.96633 Da)

Charge: +2, Monoisotopic m/z: 808.34064 Da (-2.09 mmu/-2.59 ppm), MH+: 1615.67400 Da,

RT: 49.9933 min,Identified with: Sequest HT (v1.17); XCorr:3.61,

ptmRS: Best Site Probabilities:S3(Phospho): 100, Fragment match tolerance used for search: 0.02 Da

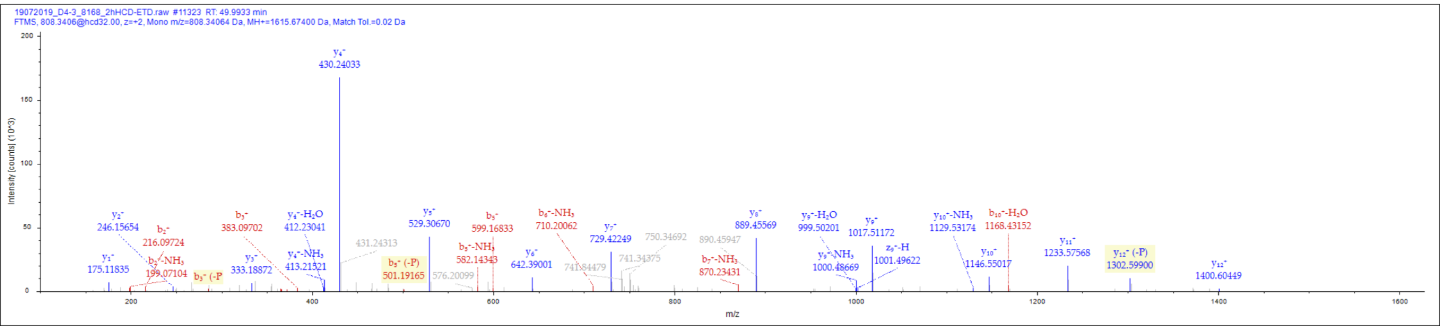

#1	b^+	b^{2+}	c^+	c^{2+}	Seq.	y^+	y^{2+}	z^+	$z{+}H^+$	$z{+}H^{2+}$	z^{2+}	$z{-}H^+$	$z{-}H^{2+}$	#2
1	129.06585	65.03657	146.09240	73.54984	Q									14
2	216.09788	108.55258	233.12443	117.06585	S	1487.61960	744.31344	1472.60870	1473.61653	737.31190	736.80799	1471.60088	736.30408	13
3	383.09624	192.05176	400.12279	200.56503	S-Phospho	1400.58757	700.79742	1385.57667	1386.58450	693.79589	693.29197	1384.56885	692.78806	12
4	470.12827	235.56777	487.15482	244.08105	S	1233.58921	617.29824	1218.57831	1219.58614	610.29671	609.79279	1217.57049	609.28888	11
5	599.17086	300.08907	616.19741	308.60234	E	1146.55718	573.78223	1131.54628	1132.55411	566.78069	566.27678	1130.53846	565.77287	10
6	727.22944	364.11836	744.25599	372.63163	Q	1017.51459	509.26093	1002.50369	1003.51152	502.25940	501.75548	1001.49587	501.25157	9
7	887.26009	444.13368	904.28664	452.64696	C-Carbami...	889.45601	445.23164	874.44511	875.45294	438.23011	437.72620	873.43729	437.22228	8
8	974.29212	487.64970	991.31867	496.16297	S	729.42536	365.21632	714.41447	715.42229	358.21478	357.71087	713.40664	357.20696	7
9	1087.37618	544.19173	1104.40273	552.70500	I	642.39334	321.70031	627.38244	628.39026	314.69877	314.19486	626.37461	313.69094	6
10	1186.44460	593.72594	1203.47114	602.23921	V	529.30927	265.15827	514.29837	515.30620	258.15674	257.65282	513.29055	257.14891	5
11	1283.49736	642.25232	1300.52391	650.76559	P	430.24086	215.62407	415.22996	416.23778	208.62253	208.11862	414.22213	207.61471	4
12	1370.52939	685.76833	1387.55594	694.28161	S	333.18809	167.09769	318.17720	319.18502	160.09615	159.59224	317.16937	159.08832	3
13	1441.56650	721.28689	1458.59305	729.80016	A	246.15607	123.58167	231.14517	232.15299	116.58013	116.07622	230.13734	115.57231	2
14					R	175.11895	88.06311	160.10805	161.11588	81.06158	80.55766	159.10023	80.05375	1

Ser711

Sequence: ESNSSCSLPSLHTSFSAPSFTAPSFLK, C6-Carbamidomethyl (57.02146 Da), S16-heavyPhospho (85.96631 Da)

Charge: +3, Monoisotopic m/z: 991.45068 Da (+3.25 mmu/+3.28 ppm), MH+: 2972.33750 Da,

RT: 61.9515 min, Identified with: Sequest HT (v1.17); XCorr:2.61,

ptmRS: Best Site Probabilities:S16(heavyPhospho): 99.28, Fragment match tolerance used for search: 0.02 Da

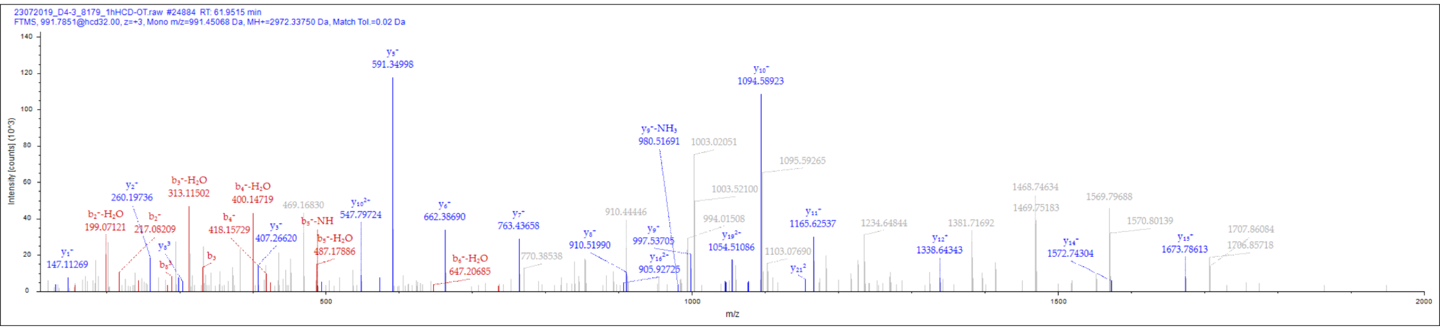

#1	b+	b2+	b3+	Seq.	y+	y2+	y3+	#2
1	130.04987	65.52857	44.02147	E				27
2	217.08190	109.04459	73.03215	S	2843.28517	1422.14622	948.43324	26
3	331.12483	166.06605	111.04646	N	2756.25314	1378.63021	919.42256	25
4	418.15685	209.58207	140.05714	S	2642.21021	1321.60874	881.40825	24
5	505.18888	253.09808	169.06781	S	2555.17818	1278.09273	852.39758	23
6	665.21953	333.11340	222.41136	C-Carbami...	2468.14615	1234.57672	823.38690	22
7	752.25156	376.62942	251.42204	S	2308.11551	1154.56139	770.04335	21
8	865.33562	433.17145	289.11673	L	2221.08348	1111.04538	741.03268	20
9	962.38839	481.69783	321.46765	P	2107.99941	1054.50334	703.33799	19
10	1049.42041	525.21385	350.47832	S	2010.94665	1005.97696	670.98707	18
11	1162.50448	581.75588	388.17301	L	1923.91462	962.46095	641.97639	17
12	1299.56339	650.28533	433.85931	H	1810.83056	905.91892	604.28170	16
13	1400.61107	700.80917	467.54187	T	1673.77165	837.38946	558.59540	15
14	1487.64310	744.32519	496.55255	S	1572.72397	786.86562	524.91284	14
15	1634.71151	817.85939	545.57535	F	1485.69194	743.34961	495.90216	13
16	1807.70985	904.35856	603.24147	S-heavyPh...	1338.62352	669.81540	446.87936	12
17	1878.74696	939.87712	626.92051	A	1165.62519	583.31623	389.21325	11
18	1975.79973	988.40350	659.27143	P	1094.58807	547.79767	365.53421	10
19	2062.83176	1031.91952	688.28210	S	997.53531	499.27129	333.18329	9
20	2209.90017	1105.45372	737.30491	F	910.50328	455.75528	304.17261	8
21	2310.94785	1155.97756	770.98747	T	763.43487	382.22107	255.14981	7
22	2381.98496	1191.49612	794.66650	A	662.38719	331.69723	221.46725	6
23	2479.03773	1240.02250	827.01743	P	591.35007	296.17868	197.78821	5
24	2566.06975	1283.53852	856.02810	S	494.29731	247.65229	165.43729	4
25	2713.13817	1357.07272	905.05091	F	407.26528	204.13628	136.42661	3
26	2826.22223	1413.61475	942.74559	L	260.19687	130.60207	87.40381	2
27				K	147.11280	74.06004	49.70912	1

Ser761

Sequence: RTSSTCSNESLNAGGTPVTPR, C6-Carbamidomethyl (57.02146 Da), S7-Phospho (79.96633 Da)

Charge: +3, Monoisotopic m/z: 758.00494 Da (-0.7 mmu/-0.92 ppm), MH+: 2272.00028 Da,

RT: 30.8200 min, Identified with: Sequest HT (v1.17); XCorr:5.88,

ptmRS: Best Site Probabilities:S7(Phospho): 100, Fragment match tolerance used for search: 0.02 Da

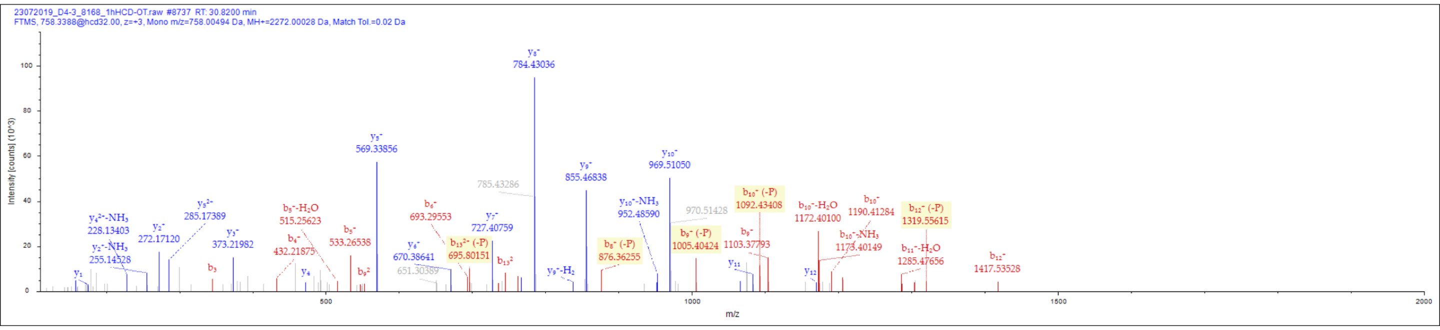

#1	b^+	b^{2+}	b^{3+}	Seq.	y^+	y^{2+}	y^{3+}	#2
1	157.10839	79.05783	53.04098	R				21
2	258.15607	129.58167	86.72354	T	2115.90125	1058.45426	705.97194	20
3	345.18809	173.09769	115.73422	S	2014.85357	1007.93043	672.28938	19
4	432.22012	216.61370	144.74489	S	1927.82155	964.41441	643.27870	18
5	533.26780	267.13754	178.42745	T	1840.78952	920.89840	614.26802	17
6	693.29845	347.15286	231.77100	C-Carbami...	1739.74184	870.37456	580.58546	16
7	860.29681	430.65204	287.43712	S-Phospho	1579.71119	790.35923	527.24191	15
8	974.33974	487.67351	325.45143	N	1412.71283	706.86005	471.57580	14
9	1103.38233	552.19480	368.46563	E	1298.66990	649.83859	433.56149	13
10	1190.41436	595.71082	397.47630	S	1169.62731	585.31729	390.54729	12
11	1303.49842	652.25285	435.17099	L	1082.59528	541.80128	361.53661	11
12	1417.54135	709.27431	473.18530	N	969.51122	485.25925	323.84192	10
13	1488.57846	744.79287	496.86434	A	855.46829	428.23778	285.82761	9
14	1545.59993	773.30360	515.87149	G	784.43118	392.71923	262.14858	8
15	1602.62139	801.81433	534.87865	G	727.40971	364.20850	243.14142	7
16	1703.66907	852.33817	568.56121	T	670.38825	335.69776	224.13427	6
17	1800.72183	900.86455	600.91213	P	569.34057	285.17392	190.45171	5
18	1899.79025	950.39876	633.93493	V	472.28781	236.64754	158.10079	4
19	2000.83792	1000.92260	667.61749	T	373.21939	187.11334	125.07798	3
20	2097.89069	1049.44898	699.96841	P	272.17172	136.58950	91.39542	2
21				R	175.11895	88.06311	59.04450	1

Ser764

Sequence: RTSSTCSNESLNAGGTPVTPR, C6-Carbamidomethyl (57.02146 Da), S7-Phospho (79.96633 Da), S10-heavyPhospho (85.96631 Da)

Charge: +2, Monoisotopic m/z: 1179.49472 Da (+6.75 mmu/+5.72 ppm), MH+: 2357.98217 Da,

RT: 53.4554 min, Identified with: Sequest HT (v1.17); XCorr:2.06,

ptmRS: Best Site Probabilities:S7(Phospho): 96.6; S10(heavyPhospho): 99.06, Fragment match tolerance used for search: 0.02 Da

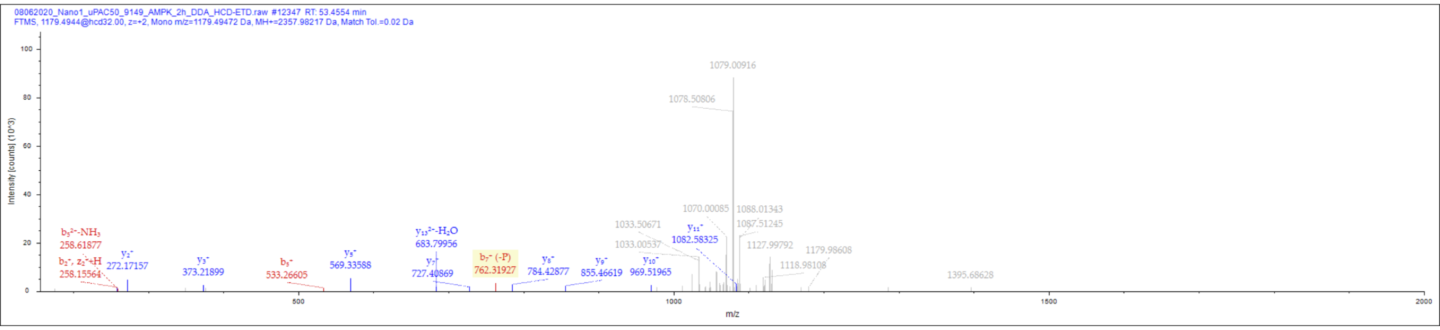

#1	b^+	b^{2+}	c^+	c^{2+}	Seq.	y^+	y^{2+}	z^+	$z{+}H^+$	$z{+}H^{2+}$	z^{2+}	$z{-}H^+$	$z{-}H^{2+}$	#2
1	157.10839	79.05783	174.13494	87.57111	R									21
2	258.15607	129.58167	275.18262	138.09495	T	2201.86756	1101.43742	2186.85666	2187.86449	1094.43588	1093.93197	2185.84884	1093.42806	20
3	345.18809	173.09769	362.21464	181.61096	S	2100.81988	1050.91358	2085.80899	2086.81681	1043.91204	1043.40813	2084.80116	1042.90422	19
4	432.22012	216.61370	449.24667	225.12697	S	2013.78786	1007.39757	1998.77696	1999.78478	1000.39603	999.89212	1997.76913	999.38820	18
5	533.26780	267.13754	550.29435	275.65081	T	1926.75583	963.88155	1911.74493	1912.75275	956.88002	956.37610	1910.73710	955.87219	17
6	693.29845	347.15286	710.32500	355.66614	C-Carbami...	1825.70815	913.35771	1810.69725	1811.70508	906.35618	905.85226	1809.68943	905.34835	16
7	860.29681	430.65204	877.32336	439.16532	S-Phospho	1665.67750	833.34239	1650.66660	1651.67443	826.34085	825.83694	1649.65878	825.33303	15
8	974.33974	487.67351	991.36629	496.18678	N	1498.67914	749.84321	1483.66824	1484.67607	742.84167	742.33776	1482.66042	741.83385	14
9	1103.38233	552.19480	1120.40888	560.70808	E	1384.63621	692.82175	1369.62532	1370.63314	685.82021	685.31630	1368.61749	684.81238	13
10	1276.38067	638.69397	1293.40722	647.20725	S-heavyPh...	1255.59362	628.30045	1240.58272	1241.59055	621.29891	620.79500	1239.57490	620.29109	12
11	1389.46473	695.23600	1406.49128	703.74928	L	1082.59528	541.80128	1067.58438	1068.59221	534.79974	534.29583	1066.57656	533.79192	11
12	1503.50766	752.25747	1520.53421	760.77074	N	969.51122	485.25925	954.50032	955.50815	478.25771	477.75380	953.49250	477.24989	10
13	1574.54477	787.77602	1591.57132	796.28930	A	855.46829	428.23778	840.45739	841.46522	421.23625	420.73233	839.44957	420.22842	9
14	1631.56624	816.28676	1648.59279	824.80003	G	784.43118	392.71923	769.42028	770.42810	385.71769	385.21378	768.41245	384.70987	8
15	1688.58770	844.79749	1705.61425	853.31076	G	727.40971	364.20850	712.39882	713.40664	357.20696	356.70305	711.39099	356.19913	7
16	1789.63538	895.32133	1806.66193	903.83460	T	670.38825	335.69776	655.37735	656.38518	328.69623	328.19231	654.36953	327.68840	6
17	1886.68814	943.84771	1903.71469	952.36098	P	569.34057	285.17392	554.32967	555.33750	278.17239	277.66847	553.32185	277.16456	5
18	1985.75656	993.38192	2002.78311	1001.89519	V	472.28781	236.64754	457.27691	458.28473	229.64601	229.14209	456.26908	228.63818	4
19	2086.80423	1043.90576	2103.83078	1052.41903	T	373.21939	187.11334	358.20850	359.21632	180.11180	179.60789	357.20067	179.10397	3
20	2183.85700	1092.43214	2200.88355	1100.94541	P	272.17172	136.58950	257.16082	258.16864	129.58796	129.08405	256.15299	128.58013	2
21					R	175.11895	88.06311	160.10805	161.11588	81.06158	80.55766	159.10023	80.05375	1

Ser789

Sequence: VASPVNK, S3-Phospho (79.96633 Da)

Charge: +2,   Monoisotopic m/z: 397.69302 Da (-1.01 mmu/-2.55 ppm),   MH+: 794.37877 Da,

RT: 20.4943 min, Identified with: Sequest HT (v1.17); XCorr:2.91,

ptmRS: Best Site Probabilities:S3(Phospho): 100, Fragment match tolerance used for search: 0.02 Da

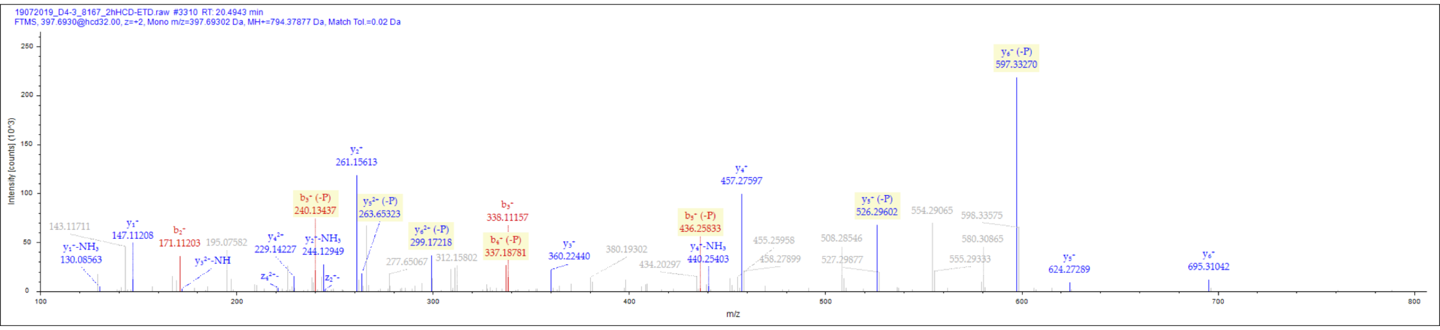

#1	b⁺	b²⁺	c⁺	c²⁺	Seq.	y⁺	y²⁺	z⁺	z+H⁺	z+H²⁺	z²⁺	z-H⁺	z-H²⁺	#2
1	100.07569	50.54148	117.10224	59.05476	V									7
2	171.11280	86.06004	188.13935	94.57331	A	695.31238	348.15983	680.30148	681.30931	341.15829	340.65438	679.29366	340.15047	6
3	338.11116	169.55922	355.13771	178.07249	S-Phospho	624.27527	312.64127	609.26437	610.27219	305.63974	305.13582	608.25654	304.63191	5
4	435.16393	218.08560	452.19048	226.59888	P	457.27691	229.14209	442.26601	443.27384	222.14056	221.63664	441.25819	221.13273	4
5	534.23234	267.61981	551.25889	276.13308	V	360.22415	180.61571	345.21325	346.22107	173.61417	173.11026	344.20542	172.60635	3
6	648.27527	324.64127	665.30182	333.15455	N	261.15573	131.08150	246.14483	247.15266	124.07997	123.57605	245.13701	123.07214	2
7					K	147.11280	74.06004	132.10191	133.10973	67.05850	66.55459	131.09408	66.05068	1

Ser794

Sequence: VASPVNKSPSAMQQQK, S8-Phospho (79.96633 Da)

Charge: +2,   Monoisotopic m/z: 890.42554 Da (-1.06 mmu/-1.19 ppm),   MH+: 1779.84380 Da,

RT: 33.6538 min, Identified with: Sequest HT (v1.17); XCorr:4.74,

ptmRS: Best Site Probabilities:S8(Phospho): 100, Fragment match tolerance used for search: 0.02 Da

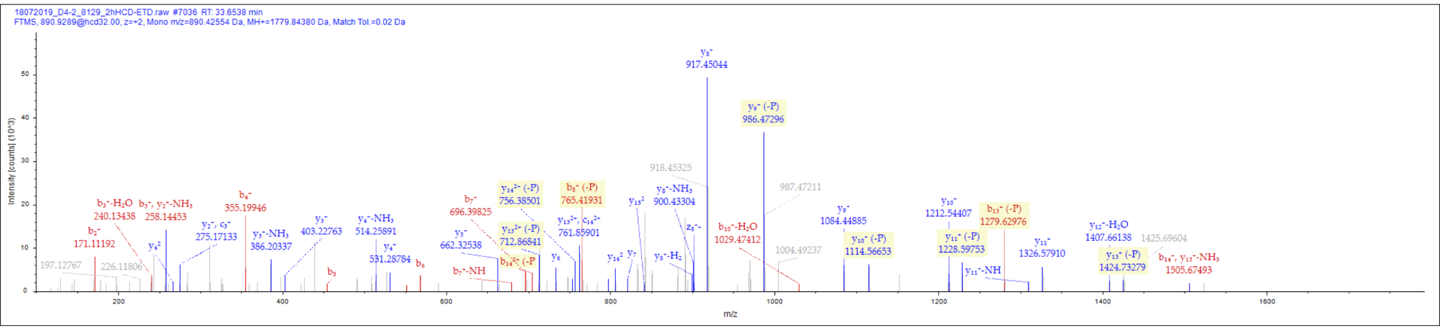

#1	b⁺	b²⁺	c⁺	c²⁺	Seq.	y⁺	y²⁺	z⁺	z+H⁺	z+H²⁺	z²⁺	z-H⁺	z-H²⁺	#2
1	100.07569	50.54148	117.10224	59.05476	V									16
2	171.11280	86.06004	188.13935	94.57331	A	1680.77750	840.89239	1665.76660	1666.77442	833.89085	833.38694	1664.75877	832.88302	15
3	258.14483	129.57605	275.17138	138.08933	S	1609.74038	805.37383	1594.72948	1595.73731	798.37229	797.86838	1593.72166	797.36447	14
4	355.19760	178.10244	372.22415	186.61571	P	1522.70835	761.85782	1507.69746	1508.70528	754.85628	754.35237	1506.68963	753.84845	13
5	454.26601	227.63664	471.29256	236.14992	V	1425.65559	713.33143	1410.64469	1411.65252	706.32990	705.82598	1409.63687	705.32207	12
6	568.30894	284.65811	585.33549	293.17138	N	1326.58718	663.79723	1311.57628	1312.58410	656.79569	656.29178	1310.56845	655.78786	11
7	696.40390	348.70559	713.43045	357.21886	K	1212.54425	606.77576	1197.53335	1198.54118	599.77423	599.27031	1196.52553	598.76640	10
8	863.40226	432.20477	880.42881	440.71804	S-Phospho	1084.44929	542.72828	1069.43839	1070.44621	535.72674	535.22283	1068.43056	534.71892	9
9	960.45502	480.73115	977.48157	489.24442	P	917.45093	459.22910	902.44003	903.44785	452.22756	451.72365	901.43220	451.21974	8
10	1047.48705	524.24716	1064.51360	532.76044	S	820.39816	410.70272	805.38726	806.39509	403.70118	403.19727	804.37944	402.69336	7
11	1118.52417	559.76572	1135.55071	568.27900	A	733.36613	367.18671	718.35524	719.36306	360.18517	359.68126	717.34741	359.17734	6
12	1249.56465	625.28596	1266.59120	633.79924	M	662.32902	331.66815	647.31812	648.32595	324.66661	324.16270	646.31030	323.65879	5
13	1377.62323	689.31525	1394.64978	697.82853	Q	531.28854	266.14791	516.27764	517.28546	259.14637	258.64246	515.26981	258.13854	4
14	1505.68181	753.34454	1522.70835	761.85782	Q	403.22996	202.11862	388.21906	389.22689	195.11708	194.61317	387.21124	194.10926	3
15	1633.74038	817.37383	1650.76693	825.88710	Q	275.17138	138.08933	260.16048	261.16831	131.08779	130.58388	259.15266	130.07997	2
16					K	147.11280	74.06004	132.10191	133.10973	67.05850	66.55459	131.09408	66.05068	1

Ser817

Sequence: DGLDRTELLPLSPLSPTMEEEPLIIFLSGDEDTEKVEEK, S15-Phospho (79.96633 Da), M18-Oxidation (15.99492 Da)

Charge: +4, Monoisotopic m/z: 1121.04089 Da (-0.97 mmu/-0.87 ppm), MH+: 4481.14174 Da,

RT: 72.5007 min, Identified with: Sequest HT (v1.17); XCorr:2.72,

ptmRS: Best Site Probabilities:S15(Phospho): 97.26, Fragment match tolerance used for search: 0.02 Da

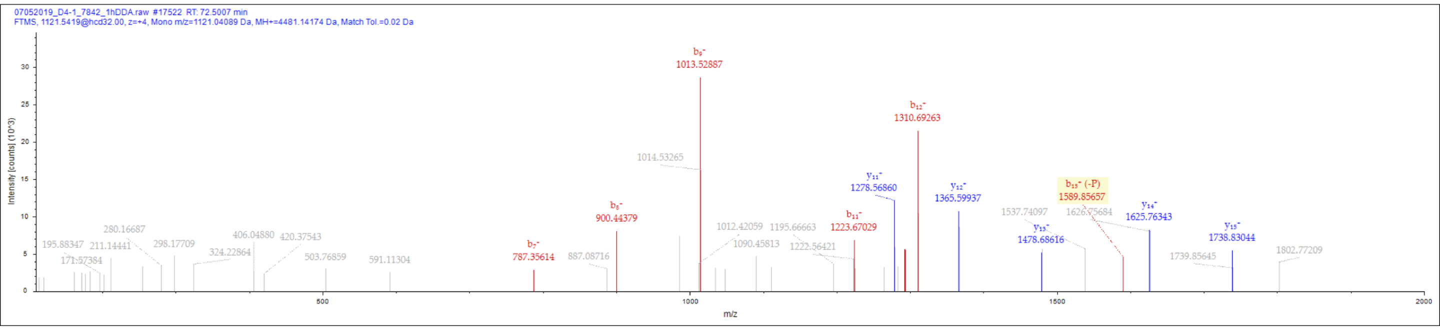

#1	b⁺	b²⁺	b³⁺	b⁴⁺	Seq.	y⁺	y²⁺	y³⁺	y⁴⁺	#2
1	116.03422	58.52075	39.34959	29.76401	D					39
2	173.05568	87.03148	58.35675	44.01938	G	4366.11869	2183.56298	1456.04441	1092.28513	38
3	286.13975	143.57351	96.05143	72.29039	L	4309.09722	2155.05225	1437.03726	1078.02976	37
4	401.16669	201.08698	134.39375	101.04713	D	4196.01316	2098.51022	1399.34257	1049.75875	36
5	557.26780	279.13754	186.42745	140.07241	R	4080.98622	2040.99675	1361.00026	1021.00201	35
6	658.31548	329.66138	220.11001	165.33433	T	3924.88511	1962.94619	1308.96655	981.97673	34
7	787.35807	394.18267	263.12421	197.59498	E	3823.83743	1912.42235	1275.28399	956.71481	33
8	900.44214	450.72471	300.81890	225.86599	L	3694.79483	1847.90106	1232.26980	924.45417	32
9	1013.52620	507.26674	338.51358	254.13701	L	3581.71077	1791.35902	1194.57511	896.18315	31
10	1110.57896	555.79312	370.86451	278.40020	P	3468.62671	1734.81699	1156.88042	867.91213	30
11	1223.66303	612.33515	408.55919	306.67121	L	3371.57394	1686.29061	1124.52950	843.64894	29
12	1310.69506	655.85117	437.56987	328.42922	S	3258.48988	1629.74858	1086.83481	815.37793	28
13	1407.74782	704.37755	469.92079	352.69241	P	3171.45785	1586.23256	1057.82413	793.61992	27
14	1520.83188	760.91958	507.61548	380.96343	L	3074.40509	1537.70618	1025.47321	769.35673	26
15	1687.83024	844.41876	563.28160	422.71302	S-Phospho	2961.32102	1481.16415	987.77853	741.08571	25
16	1784.88301	892.94514	595.63252	446.97621	P	2794.32266	1397.66497	932.11241	699.33612	24
17	1885.93069	943.46898	629.31508	472.23813	T	2697.26990	1349.13859	899.76148	675.07293	23
18	2032.96609	1016.98668	678.32688	508.99698	M-Oxidation	2596.22222	1298.61475	866.07892	649.81101	22
19	2162.00868	1081.50798	721.34108	541.25763	E	2449.18682	1225.09705	817.06713	613.05216	21
20	2291.05127	1146.02927	764.35527	573.51828	E	2320.14423	1160.57575	774.05293	580.79151	20
21	2420.09386	1210.55057	807.36947	605.77892	E	2191.10164	1096.05446	731.03873	548.53087	19
22	2517.14663	1259.07695	839.72039	630.04211	P	2062.05904	1031.53316	688.02453	516.27022	18
23	2630.23069	1315.61898	877.41508	658.31313	L	1965.00628	983.00678	655.67361	492.00703	17
24	2743.31476	1372.16102	915.10977	686.58415	I	1851.92222	926.46475	617.97892	463.73601	16
25	2856.39882	1428.70305	952.80446	714.85516	I	1738.83815	869.92271	580.28423	435.46500	15
26	3003.46723	1502.23726	1001.82726	751.62227	F	1625.75409	813.38068	542.58955	407.19398	14
27	3116.55130	1558.77929	1039.52195	779.89328	L	1478.68567	739.84648	493.56674	370.42688	13
28	3203.58333	1602.29530	1068.53263	801.65129	S	1365.60161	683.30444	455.87205	342.15586	12
29	3260.60479	1630.80603	1087.53978	815.90665	G	1278.56958	639.78843	426.86138	320.39785	11
30	3375.63173	1688.31950	1125.88210	844.66339	D	1221.54812	611.27770	407.85422	306.14249	10
31	3504.67433	1752.84080	1168.89629	876.92404	E	1106.52117	553.76423	369.51191	277.38575	9
32	3619.70127	1810.35427	1207.23861	905.68077	D	977.47858	489.24293	326.49771	245.12510	8
33	3720.74895	1860.87811	1240.92117	930.94269	T	862.45164	431.72946	288.15540	216.36837	7
34	3849.79154	1925.39941	1283.93536	963.20334	E	761.40396	381.20562	254.47284	191.10645	6
35	3977.88650	1989.44689	1326.63369	995.22708	K	632.36137	316.68432	211.45864	158.84580	5
36	4076.95492	2038.98110	1359.65649	1019.99419	V	504.26640	252.63684	168.76032	126.82206	4
37	4205.99751	2103.50239	1402.67069	1052.25483	E	405.19799	203.10263	135.73751	102.05496	3
38	4335.04010	2168.02369	1445.68489	1084.51548	E	276.15540	138.58134	92.72332	69.79431	2
39					K	147.11280	74.06004	49.70912	37.53366	1
