# Supplementary material for: AKT/AMPK-mediated phosphorylation of TBC1D4 disrupts the interaction with insulin-regulated aminopeptidase
Source: J Biol Chem. 2021 Apr 16;296:100637. doi: 10.1016/j.jbc.2021.100637 (PMC8131924; doi:10.1016/j.jbc.2021.100637)
Supplement: Supporting information [file mmc2.pdf]

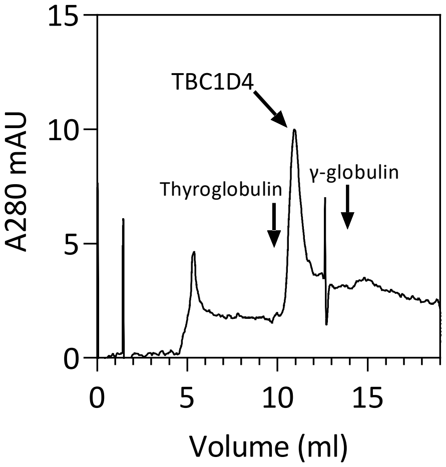

Figure S1. Representative chromatogram of purified TBC1D4 on a SEC column. Proteins were separated by fast protein LC (FPLC) with an ENrich SEC650 column (Bio-Rad) in 50 mM Tris-HCl pH 7.5 at a flow rate of 1 ml/min and the absorption was measured at 280 nM. Arrows indicate elution positions of standard proteins, bovine thyroglobulin (670,000 Da) and bovine γ-globulin (158,000 Da).

A

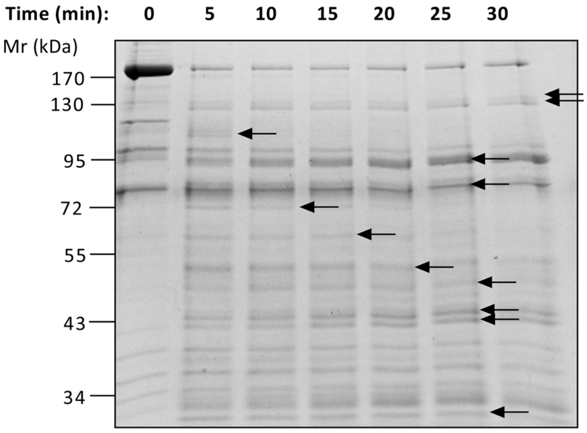

B

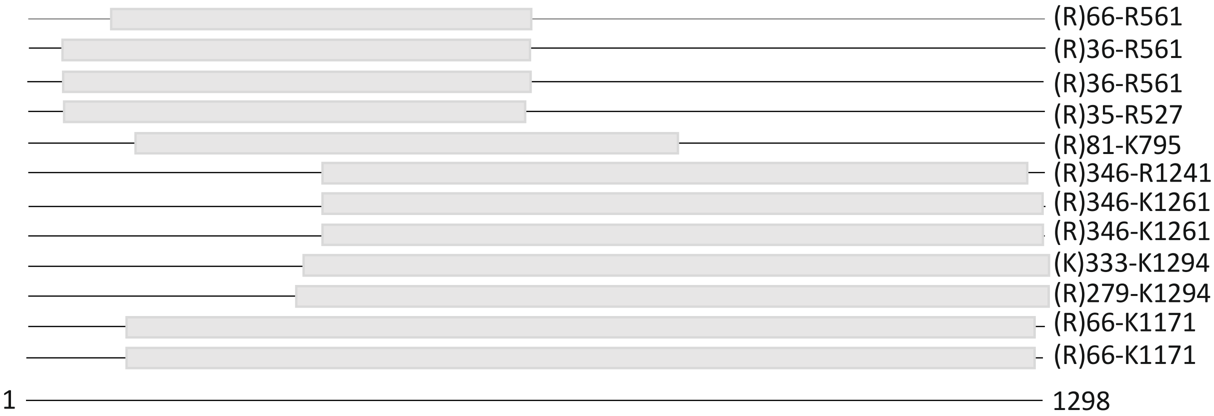

Figure S2. Limited proteolysis of TBC1D4. A, 0.2 µg TBC1D4 was incubated with 125 ng of trypsin in a volume of 25 µl. The reaction was stopped at different time points by adding SDS-sample buffer and samples were separated by SDS-PAGE. Coomassie-stained bands (marked by arrows) were excised and subjected to in-gel protease digest and the resulting peptides were analyzed by mass spectrometry. B, alignment of TBC1D4 peptides identified with high confidentiality using Proteome Discoverer^{TM} 2.3 software. Numbers indicate positions of N- and C-terminal amino acids in the full-length protein. To define robust assignment of protein fragment sequences after partial digest, only peptides identified with at least 5 peptide spectrum matches (PSMs) were considered.

For MS analysis of partial digest samples, resulting peptides from in-gel digest were reconstituted in 1% TFA (v/v) supplemented with iRT peptides (Biognosys) and separated by liquid chromatography (Ultimate 3000, ThermoFisher Scientific) using an EASYspray ion source equipped to an Orbitrap Fusion Lumos mass spectrometer (ThermoFisher Scientific). Peptides were trapped and desalted on an Acclaim PepMap C18-LC-column (2 cm; 164535 ThermoFisher Scientific) and subsequently separated via EASY-spray C18 column (50 cm; ES803 ThermoFisher Scientific) using the following gradient: Peptides were loaded with a flow rate of 6 µl/min, to the trap column (loading buffer: (0.1% TFA (v/v)). After 4 min, trap column was switched into nano-flow (300 nl/min) and peptides were eluted through the separation column with buffer A (0.1% FA (v/v)) using a linear gradient 4-34% buffer B (0.1% FA, 80% ACN (v/v)) for 30 min, followed by a 5 min increase to 50% buffer B, a 1 min increase to 90% buffer

B and a 10 min wash with buffer B at a flow rate of 300 nl/min. Column temperature was set to 40°C. MS data were acquired with data-dependent (DDA) scan methods using an Orbitrap for fullscans (MS1), resolution of 120,000, scan range 350-1600 m/z, AGC target of $4.0e^5$. For MS2 the TOP20 precursors with an intensity threshold of $5e^3$ and a charge state from 2-7 were fragmented with HCD (higher-energy C-trap dissociation, collision energy 32%) and detected in the ion-trap with rapid scan rate mode, an AGC target of $1e^4$ and a maximum injection time of 50 ms. Mass spectrometry raw files were analyzed with Proteome Discoverer™ 2.3 software (ThermoFisher Scientific). HTsequest search was done against FASTA files (SwissProt Mus musculus (TaxID=10090, version 2017-10-25, 25097 sequences), a general contaminants list (245 sequences) and His-tagged TBC1D4 sequence), enzyme was set to trypsin with maximum 2 missed cleavage sites allowed, and for HCD fragmentation b and y Ions were selected with a fragment mass tolerance of 0.6 Da. Precursor mass tolerance 10 ppm. Carbamidomethylation of cysteine was set as fixed modification. N-terminal acetylation and methionine oxidation were allowed as variable modifications. The fixed value PSM Validator (max delta Cn: 0.05) were applied. Only high confident peptides were annotated to proteins. To define robust assignment of protein fragment sequences, identified peptides were filtered according to PSM counts. Only peptides, identified with at least 5 peptide spectrum matches (PSM's) were considered to compose protein fragments.

The mass spectrometry proteomics data have been deposited to the ProteomeXchange Consortium via the PRIDE (49) partner repository with the dataset identifiers PXD024645.

Table S1. Analysis of TBC1D4 phosphorylation sites via mass spectrometry.

Residue Position	Annotated Sequence	Precursor Mass [m/z]	Charge State	HTSequest Score [Xcorr]	Modifications	ptmRS: for indicated phosphosite
Ser^{258}	GGDPGDEMGVLEVESPVSPDDSL PEK	1376.5820	+2	2.56	**S15-Phospho** (79.9663 Da)<br>M8-Oxidation (15.9949 Da)	100
Ser^{261}	GGDPGDEMGVLEVESPVSPDDSL PEKADGTVNSPR	1211.8689	+3	6.5	**S18-Phospho** (79.9663 Da)	99.9
Ser^{276}	ADGTVNSPR	498.7099	+2	3.1	**S7-Phospho** (79.9663 Da)	100
Ser^{324}	CSSVTGVMQK	591.7481	+2	2.32	**S3-heavyPhospho** (85.9663 Da)<br>C1-Carbamidomethyl (57.02146 Da)	100
Ser^{348}	HASAPSHVQPSDSEKNR	483.9694	+4	2.81	**S3-heavyPhospho** (85.9663 Da)	100
Ser^{377}	FEINLISPDTK	678.8242	+2	2.55	**S7-Phospho** (79.9663 Da)	100
Ser^{492}	HLSSLTDNEQADIFER	978.7336	+2	3.64	**S3-Phospho** (79.9663 Da)	100
Ser^{577}	SLTSSLENIFSR	720.3439	+2	2.25	**S5-heavyPhospho** (85.9663 Da)	99.65
Ser^{595}	GRLGSMDSFER	447.5273	+3	2.8	**S5-heavyPhospho** (85.9663 Da)	100
Ser^{598}	LGSMDSFER	561.2192	+2	3.13	**S6-Phospho** (79.9663 Da)	100
Thr^{649}	AHTFSHPPSSSR	466.2053	+3	3.51	**T3-heavyPhospho** (85.9663 Da)	100
Ser^{673}	AHGLRSPLLR	400.5511	+3	3.63	**S6-Phospho** (79.9663 Da)	100
Ser^{680}	QSSSEQCSIVPSAR	808.3406	+2	3.61	**S3-Phospho** (79.9663 Da)<br>C7-Carbamidomethyl (57.02146 Da)	100
Ser^{711}	ESNSSCSLPSLHTSFsAPSFTAPSFL K	991.7851	+3	2.61	**S16-heavyPhospho** (85.9663 Da)<br>C6-Carbamidomethyl (57.02146 Da)	99.28
Ser^{761}	RTSSTCsNESLNAGGTPVTPR	758.3388	+3	5.88	**S7-Phospho** (79.9663 Da)<br>C6-Carbamidomethyl (57.02146 Da)	100
Ser^{764}	RTSSTCSNEsLNAGGTPVTPR	1179.4944	+2	2.06	**S10-heavyPhospho** (85.9663 Da)<br>S7-Phospho (79.9663 Da)<br>C6-Carbamidomethyl (57.02146 Da)	99.06
Ser^{789}	VASPVNK	397.6930	+2	2.91	**S3-Phospho** (79.9663 Da)	100
Ser^{794}	VASPVNKsPSAMQQQK	890.9289	+2	4.74	**S8-Phospho** (79.9663 Da)	100
Ser^{817}	DGLDRTELLPLSPLsPTMEEEPLIIF LSGDEDTEKVEEK	1121.5419	+4	2.72	**S15-Phospho** (79.9663 Da)<br>M18-Oxidation (15.9949 Da)	97.26

Peptides were trapped and desalted on an Acclaim PepMap™ C18 column (2 cm length; 164535 ThermoFisher Scientific) and subsequently separated via EASY-Spray™ C18 column (50 cm; ES803A ThermoFisher Scientific) and µPAC™ (50 cm; 552503518050, Pharmafluidics) using two different gradients:

1. For 90 min LC-MS run, peptides were loaded with a flow rate of 6 µl/min, to the trap column (loading buffer: (0.1% TFA (v/v)). After 4 min, trap column was switched into nano flow (300 nl/min) and peptides were eluted through the separation column with buffer A (0.1% FA (v/v)) using a linear gradient 4-34% buffer B (0.1% FA, 80% ACN (v/v)) for 55 min, followed by a 10 min increase to 50% buffer B, a 1 min increase to 90% buffer B and a 10 min wash with buffer B at a flow rate of 300 nl/min.

2. For 150 min LC-MS run, the same loading procedure was used, but the linear gradient from 4-34% buffer B (0.1% FA, 80% ACN (v/v)) lasts 100 min, followed by a 20 min increase to 50% buffer B, a 1 min increase to 90% buffer B and a 10 min wash with buffer B at a flow rate of 300 nl/min.

Column temperature was set to 40°C. MS data were acquired with data-dependent (DDA) scan methods using an Orbitrap for fullscans (MS1), resolution of 120,000, scan range 350-1600 m/z, AGC target of $4.0e^5$ and cycle time of 3 seconds.
Filter criteria and scan parameter for MS2 were set as follows:

1. For 90min LC-MS runs precursors with an intensity threshold of $2.5e^4$ and a charge state from 2-7 were selected for MS2 fragmentation with HCD (higher-energy C-trap dissociation; collision energy 32%). Detection in the Orbitrap was done with an AGC target of $1e^5$ and a maximum injection time of 54 ms at 30,000 resolution.

2. For 150min LC-MS runs two fragmentation methods were used. Precursor with an intensity threshold greater than $2.5e^4$ were selected for MS2. Those with a charge state of 2 were fragmented with HCD (32%) and those with charge states 3-7 with ETD (electron transfer dissociation). Cycle time between two full scans were set to 3 sec and with both fragmentation methods an AGC target of $1e^5$, a maximum injection time of 150 ms and Orbitrap resolution of 15,000 was set.

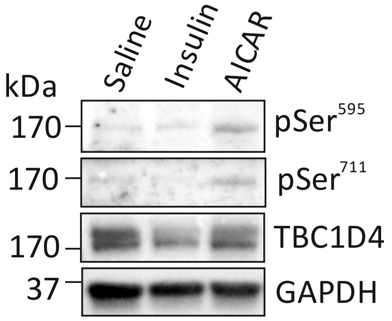

Figure S3. ***In vivo*** **TBC1D4 phosphorylation.** Male C57BL/6J mice were injected with either saline, 1 IU/kg insulin or 250 mg/kg AICAR and sacrificed by cervical dislocation after 60 minutes. Tissues were removed and immediately frozen in liquid nitrogen. Protein lysates from stimulated and unstimulated Gastrocnemius muscles were prepared, separated by SDS-PAGE and subjected to Western Blot analysis for AKT- and AMPK phosphorylation using TBC1D4 phosphospecific antibodies against $pSer^{595}$ and $pSer^{711}$, TBC1D4 and GAPDH.

A

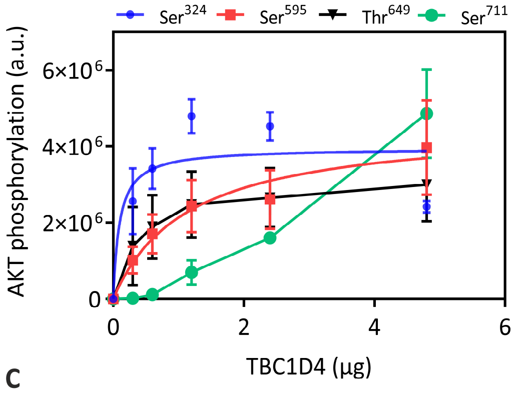

B

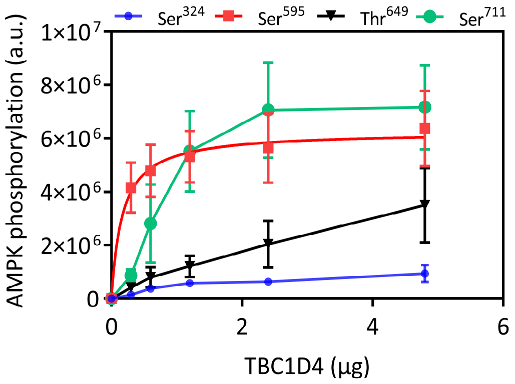

C

	Vmax (a.u.)	
	AKT	AMPK
Ser^{324}	3.9×10^6	1.1×10^6
Thr^{649}	3.1×10^6	8.9×10^6
Ser^{595}	4.5×10^6	6.2×10^6
Ser^{711}	[illegible]	9.8×10^6

Figure S4. Kinetics of TBC1D4 phosphorylation by AKT and AMPK *in vitro*. A, B, purified AKT and AMPK were used to phosphorylate purified TBC1D4 (between 0.3 and 4.8 µg) for 5 min at RT. Phosphorylation of TBC1D4 was confirmed using phosphosite-specific antibodies against Ser^{324}, Thr^{649} $Ser^{595}$and Ser^{711} and the data were then subjected to non-linear curve fitting to Michaelis-Menten kinetics using GraphPad Prism Software. C, apparent V_{max} values for individual phosphorylation sites. Data represent mean values ± SEM from four independent experiments.

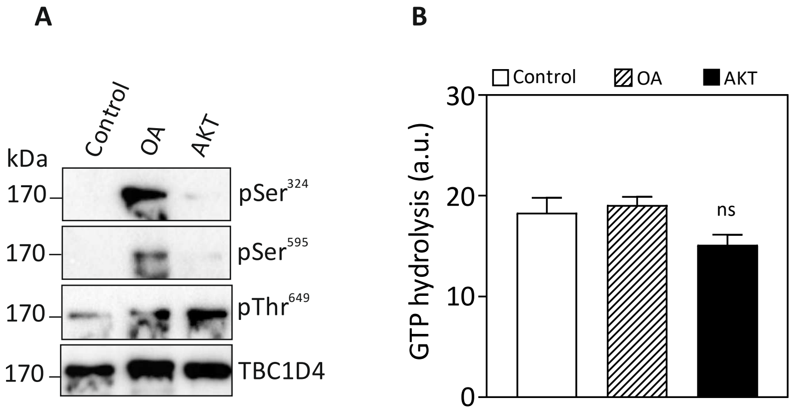

Figure S5. Impact of phosphorylation on TBC1D4 GAP activity *in vivo*. Baculovirus-infected *Sf*9 cells expressing TBC1D4 were treated with okadiac acid (OA; 50 nmol for 30 minutes) before harvest. In addition, *Sf*9 cells were co-transfected with baculovirus expressing TBC1D4 and constitutively active AKT (MOI TBC1D4: AKT; approx. 7:5) and harvested three days post infection. The cells were lysed and full-length TBC1D4 was purified as described in "Experimental procedures". A, immunoblot analysis of purified TBC1D4 using phosphosite-specific antibodies against Ser^{324}, Ser^{595} and Thr^{649}. B, affinity-purified GST-Rab10 (0.6–1 pmol) loaded with [γ-^{32}P]GTP was incubated in the absence or presence of 3 pmol of purified phosphorylated TBC1D4 as described under "Experimental procedures." After 30 min at room temperature (RT), aliquots were filtrated through activated charcoal and radioactive [^{32}P]phosphate was determined by scintillation counting. Data represent mean values ± SEM from three experiments; ns, not significant.
